# Electrostatic interactions at the five-fold axis alter heparin-binding phenotype and drive enterovirus A71 virulence in mice

**DOI:** 10.1371/journal.ppat.1007863

**Published:** 2019-11-15

**Authors:** Han Kang Tee, Chee Wah Tan, Thinesshwary Yogarajah, Michelle Hui Pheng Lee, Hann Juang Chai, Nur Aziah Hanapi, Siti R. Yusof, Kien Chai Ong, Vannajan Sanghiran Lee, I-Ching Sam, Yoke Fun Chan

**Affiliations:** 1 Department of Medical Microbiology, Faculty of Medicine, University of Malaya, Kuala Lumpur, Malaysia; 2 Department of Pharmacology, Faculty of Medicine, University of Malaya, Kuala Lumpur, Malaysia; 3 Centre for Drug Research, Universiti Sains Malaysia, Penang, Malaysia; 4 Department of Biomedical Science, Faculty of Medicine, University of Malaya, Kuala Lumpur, Malaysia; 5 Department of Chemistry, Faculty of Science, University of Malaya, Kuala Lumpur, Malaysia; National Institute of Infectious Diseases, JAPAN

## Abstract

Enterovirus A71 (EV-A71) causes hand, foot and mouth disease epidemics with neurological complications and fatalities. However, the neuropathogenesis of EV-A71 remains poorly understood. In mice, adaptation and virulence determinants have been mapped to mutations at VP2-149, VP1-145 and VP1-244. We investigate how these amino acids alter heparin-binding phenotype and shapes EV-A71 virulence in one-day old mice. We constructed six viruses with varying residues at VP1-98, VP1-145 (which are both heparin-binding determinants) and VP2-149 (based on the wild type 149K/98E/145Q, termed KEQ) to generate KKQ, KKE, KEE, IEE and IEQ variants. We demonstrated that the weak heparin-binder IEE was highly lethal in mice. The initially strong heparin-binding IEQ variant acquired an additional mutation VP1-K244E, which confers weak heparin-binding phenotype resulting in elevated viremia and increased virus antigens in mice brain, with subsequent high virulence. IEE and IEQ-244E variants inoculated into mice disseminated efficiently and displayed high viremia. Increasing polymerase fidelity and impairing recombination of IEQ attenuated the virulence, suggesting the importance of population diversity in EV-A71 pathogenesis *in vivo*. Combining *in silico* docking and deep sequencing approaches, we inferred that virus population diversity is shaped by electrostatic interactions at the five-fold axis of the virus surface. Electrostatic surface charges facilitate virus adaptation by generating poor heparin-binding variants for better *in vivo* dissemination in mice, likely due to reduced adsorption to heparin-rich peripheral tissues, which ultimately results in increased neurovirulence. The dynamic switching between heparin-binding and weak heparin-binding phenotype *in vivo* explained the neurovirulence of EV-A71.

## Author summary

Enterovirus A71 (EV-A71) is the primary cause of hand, foot and mouth disease, and it can also infect the central nervous system and cause fatal outbreaks in young children. EV-A71 pathogenesis remains elusive. In this study, we demonstrated that EV-A71 variants with strong affinity to heparan sulfate (heparin) have a growth advantage in cell culture, but are disadvantaged *in vivo*. When inoculated into one-day old mice, strong heparin-binding virus variants are more likely to be adsorbed to peripheral tissues, resulting in impaired ability to disseminate, and are cleared from the bloodstream rapidly. The lower viremia level resulted in no neuroinvasion. In contrast, weak heparin-binding variants show greater levels of viremia, dissemination and subsequent neurovirulence in mice. We also provide evidence that the EV-A71 heparin-binding pattern is mediated by electrostatic surface charges on the virus capsid surface. In mice, EV-A71 undergoes adaptive mutation to acquire greater negative surface charges, thus generating new virulent variants with weak heparin-binding ability which allows greater viral spread. Our study underlines the importance of electrostatic surface charges in shaping EV-A71 virulence.

## Introduction

Enterovirus A71 (EV-A71) causes cyclical outbreaks of hand, foot and mouth disease (HFMD) in the Asia-Pacific region [1]. HFMD primarily affects children younger than 5 years old. The clinical manifestations are usually mild and characterized by fever, oral ulcers and skin rashes on hands and feet [2, 3]. In some cases, infection also results in severe neurological complications, including encephalitis, aseptic meningitis, acute flaccid paralysis and death [4]. There are no licensed antivirals, and licensed vaccines are only available in China. The EV-A71 genome encodes a polyprotein with a single open reading frame (ORF) flanked by 5’ and 3’ untranslated regions [5]. The polyprotein is cleaved into four capsid proteins (VP1 to VP4) and seven nonstructural proteins (2A, 2B, 2C and 3A to 3D). The capsid proteins form a protomer, and five protomers form a pentamer, and then twelve pentamers assemble around a genome forming a provirion. The five-fold axis symmetry is formed by VP1 and surrounded by a canyon. Virus-receptor binding at the canyon or other physical alterations, such as heat, will displace the lipid pocket factor and trigger viral uncoating [6].

Multiple receptors including human scavenger receptor class B2 (SCARB2) [7], P-selectin glycoprotein ligand-1 (PSGL-1) [8], heparan sulfate [9], sialylated glycan [10, 11], annexin II [12], vimentin [13] and nucleolin [14] have roles in EV-A71 attachment or entry. Following infection, EV-A71 can disseminate to different organs and invade the human central nervous system (CNS) either through retrograde axonal transport or hematogenous spread [15–18]. However, the neuropathogenesis of EV-A71 remains unclear and virulence determinants are not well elucidated. Recent reports have shown that EV-A71 strains with VP1-145G/Q were more frequently isolated from severe HFMD with neurological complications [19–23]. However, the results contradicted *in vivo* studies which showed that VP1-145E strains but not 145G/Q exhibit higher virulence and lethality in murine models [24–27]. These findings were also consistent with studies in cynomolgus monkeys, in which strong selection of VP1-145E over VP1-145G was observed [28]. VP1-145G/Q residues but not VP1-145E are responsible for binding to the receptor PSGL-1 [29]. Both VP1-145E and VP2-149I mutations conferred Chinese Hamster Ovary (CHO) cell adaptation and increased mouse virulence [24, 26, 30].

Neurotropism and neurovirulence have been previously related to the virus binding to heparan sulfate (heparin), a negatively-charged glycosaminoglycan (GAG) found abundantly on most cell surfaces. Strong affinity for heparin was reported to cause attenuation in viruses such as Theiler's murine encephalomyelitis virus [31], Japanese encephalitis virus [32], Murray Valley encephalitis virus [32], West Nile virus [33], yellow fever virus [34] and tick-borne encephalitis virus [35]. In contrast, virus variants with weak heparin-binding resulted in higher mortality in mice, as shown for Sindbis virus [36] and eastern equine encephalitis virus (EEEV) [37]. Interestingly, variants with strong heparin-binding can contribute to higher neurovirulence if inoculated directly into the CNS, as demonstrated with EEEV [38].

EV-A71 also utilizes heparin as an attachment receptor [9]. Amino acids clustered around the five-fold symmetry axis, specifically VP1-98, VP1-145, VP1-242 and VP1-244 modulate positive charges required for heparin-binding [39]. The VP1-145G/Q residues are associated with stronger heparin-binding, but not negatively-charged VP1-145E. The propensity of EV-A71 to acquire positively-charged residues at the five-fold axis is associated with increased heparin-binding upon *in vitro* culture adaptation [39]. In contrast, experimental studies of VP1-145E in mice and monkey studies showed higher virulence and lethality.

In this current study, we sought to delineate the discrepancy exhibited between *in vitro* cytopathogenicity due to heparin-binding and association of weak heparin-binding with *in vivo* virulence. We demonstrated that acquisition of negative charges at the five-fold axis reduced heparin-binding. This resulted in high viremia and enhanced lethality in mice.

## Results

### Construction and rescue of clone-derived virus variants

**Fig 1:**
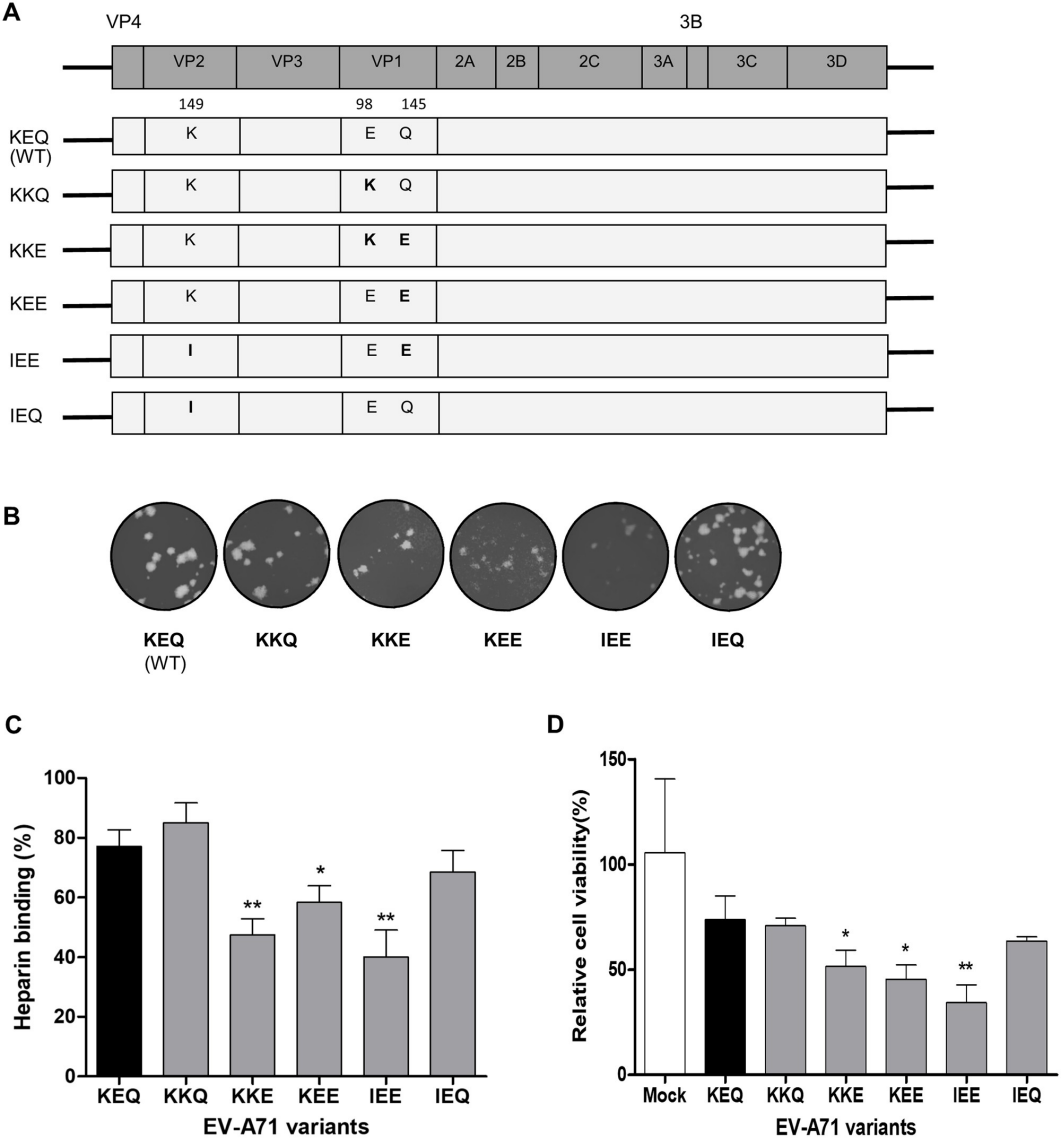
*In vitro* characterization of constructed EV-A71 variants. (A) Schematic illustration of the EV-A71 genome and the infectious clone constructs. Different amino acids were substituted at VP2-149, VP1-98 and VP1-145 (labeled in bold), with reference to the wild type (WT) strain KEQ. (B) The clone-derived EV-A71 variants were propagated in RD cells and showed comparable plaque morphologies (C) The binding affinity of EV-A71 variants to heparin sepharose beads was analyzed. (D) Inhibitory effect of heparin on EV-A71 variants was evaluated by pretreating the viruses with soluble heparin before infection of RD cells. Results are presented as mean ± SD (n = 3). Error bars indicate standard deviations from triplicates. Statistical significances are denoted with *P < 0.05, **P < 0.01 as compared to the WT.

We have previously described the role of VP1-98 and VP1-145 as modulators of heparin-binding in cell culture [39]. In the present study, we elucidated the role of heparin-binding in shaping EV-A71 virulence in mice. Five EV-A71 variants were engineered from a laboratory-adapted EV-A71 strain (5865/SIN/00009, subgenogroup B4), which posesses VP2-149K, VP1-98E and VP1-145Q residues (designated as KEQ, following amino acid residue order). Site-directed mutagenesis was performed to generate the five variants denoted as KKQ, KKE, KEE, IEE and IEQ (Fig 1A). Some variants had the mouse-adaptive determinant, K149I [24, 26, 40] along with substitutions in the heparin-binding determinants, VP1-98 and VP1-145. After a single passage, all EV-A71 variants were viable with comparable plaque morphology (Fig 1B) and were confirmed by sequencing (S1 Fig).

### Heparin-binding phenotypes of EV-A71 in *in vitro* cell culture

We further investigated the heparin-binding of the EV-A71 variants. EV-A71 variants with VP1-145E (KKE, KEE and IEE) displayed significant reduction of 29.7%, 18.8% and 37.1% in heparin-binding, respectively (Fig 1C). Similar findings were also observed in a heparin inhibition assay. Pre-treated heparin inhibition of these three VP1-145E variants significantly reduced RD cell viability to 51.5%, 45.5% and 34.3%, respectively (Fig 1D). Based on these heparin-binding results, the EV-A71 variants were categorized into two groups: strong heparin binder (KEQ, KKQ and IEQ) and weak heparin binder (KKE, KEE and IEE).

### Potential association of weak heparin-binding variants with *in vivo* virulence

**Fig 2:**
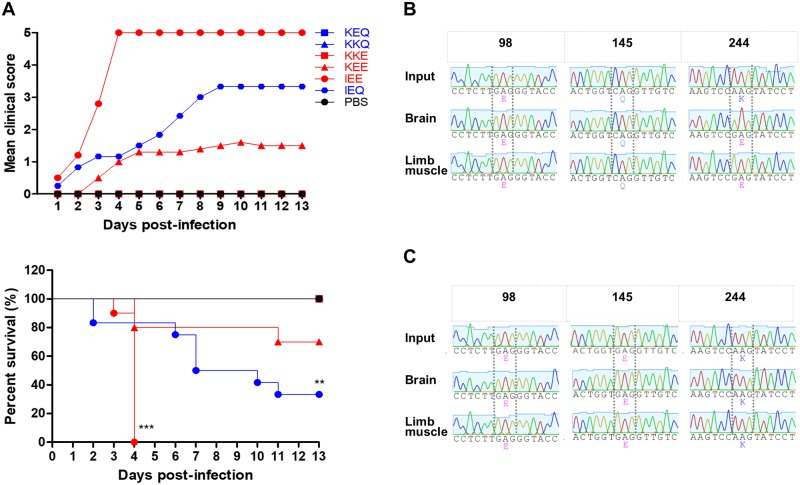
Clinical scores and survival rates of suckling mice infected with EV-A71 variants. One-day old suckling mice (n = 9–12) were inoculated with 1 × 10^5^ PFU of different EV-A71 variants by i.p. injection. (A) A litter of mock-infected mice was used as a control group receiving PBS injection. The clinical scores and percentage of survival of the infected mice groups were monitored daily for 13 days. The severity of clinical symptoms was scored as follows: 0, healthy; 1, weak or less active; 2 hunched posture and lethargy; 3, one-limb paralysis; 4, two-limb paralysis; 5, moribund or dead. Significant differences compared to KEQ are labelled as * (*P* < 0.05) and ***P (< 0.001). KEQ, KKQ and KKE curves are identical to that of the mock-infected group. The representative VP1 sequence chromatograms of IEQ (B) and IEE (C) populations isolated from infected animal organs (n = 3) are shown, highlighting VP1-98, VP1-145 and VP1-244. The emergence of IEQ-244E virus isolated from brains and limbs of IEQ-infected moribund mice (n = 3) are shown.

To determine the role of heparin-binding in EV-A71 *in vivo* virulence, we performed intraperitoneal (i.p.) infection of one-day old suckling mice with 1 × 10^5^ PFU of each EV-A71 variant. The clinical score and survival analysis of infected mice are shown in Fig 2A. IEE-infected mice exhibited the highest virulence *in vivo*, with all the infected mice dying by day 4 post-infection (n = 10). Notably, 66.7% (n = 8) of the IEQ-infected mice died by day 12 post-infection, followed by 20% (n = 2) mortality in KEE-infected mice. Consistent with previous findings [24–26, 41], EV-A71 variants with VP1-145E were associated with an increased virulence phenotype in animal models, except for KKE. Mice infected with other EV-A71 variants showed no apparent clinical signs and survived beyond day 13 post-infection. These data suggest that *in vivo* pathogenicity in mice was increased by an additional VP2-K149I mutation, as demonstrated in the IEE-infected mice.

We further asked why the IEQ variant, a strong heparin binder, exhibited a relatively high virulence in mice. Viral genomic RNA from the brains and hind limbs of dead IEQ-infected mice (n = 3) were harvested for genome sequencing, and revealed that the IEQ variant had acquired a VP1-K244E mutation (Fig 2B). This mutation however, was not present in the IEE-infected mice (Fig 2C).

### Emergence of IEQ-K244E variant resulted in abolished heparin-binding and regained virulence

**Fig 3:**
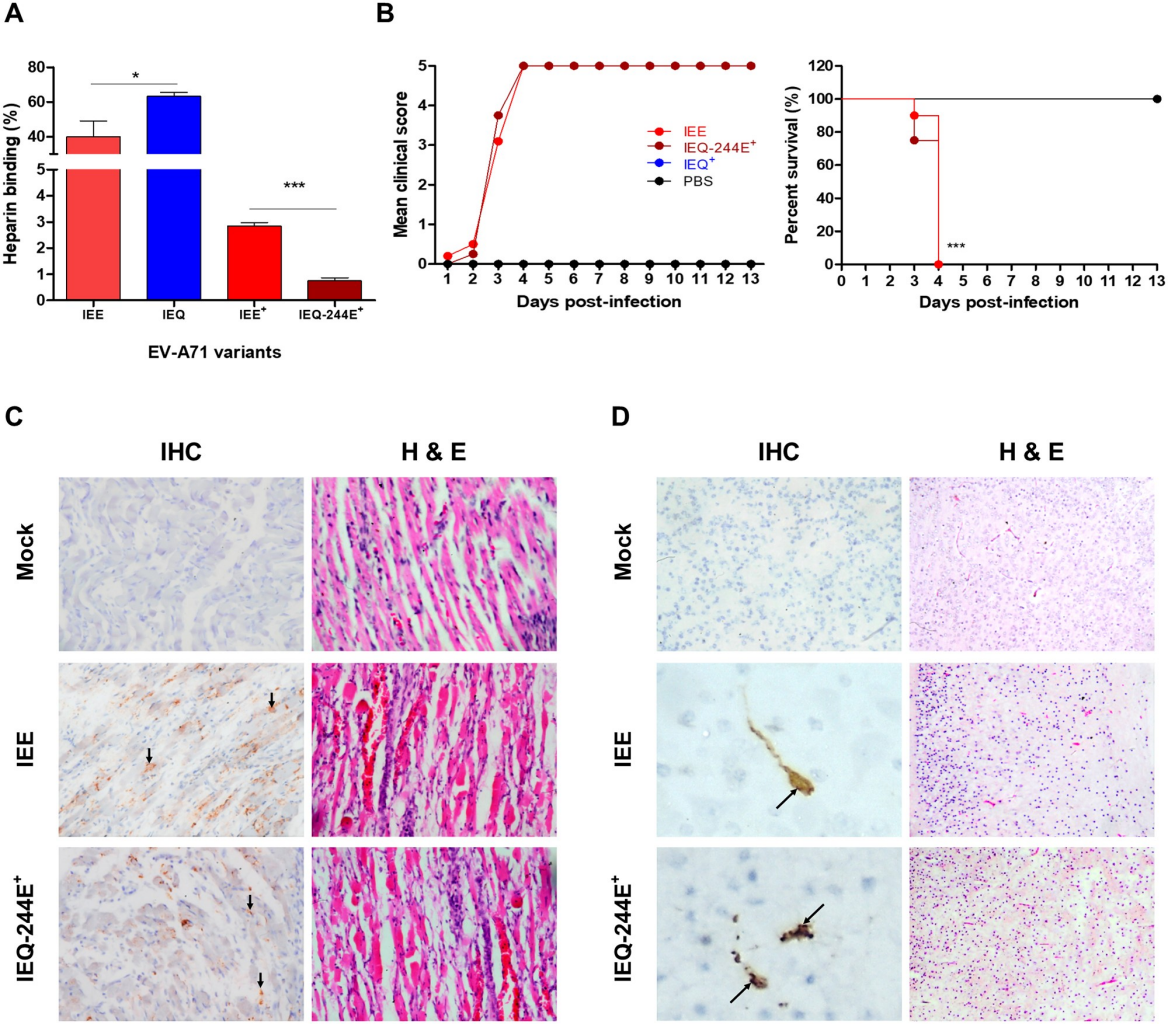
Characterization of virulent phenotype of IEE and IEQ-244E^+^ variants. (A) EV-A71 variants collected from the brain homogenates of dead infected mice were compared to clone-derived variants for heparin-binding properties. (B) One-day old suckling mice (n = 8–12) were infected with IEE, IEQ-244E^+^ and IEQ^+^ through i.p. injection. A control group receiving PBS injection was also included. The clinical scores and percentage of survival are shown over 13 days post-infection. Significant differences compared to WT are labelled as ** (*P* < 0.01) and ***P (< 0.001). Tissue samples of mice which succumbed to IEE (n = 2) and IEQ-244E^+^ (n = 2) infection were subjected to IHC and H&E staining. Virus antigen was seen in muscle cells (C), along with increased inflammatory infiltrates. In brains (D), antigen-positive neurons were seen in the pons (representative image from an IEE-infected brain) and dentate nucleus (representative image from an IEQ -244E^+^-infected brain), with mild inflammation in the cortex shown by H&E staining. Magnification for IHC staining: X40; H&E staining: X20.

Since VP1-244K is a key determinant of heparin-binding [39], we speculated that the emergence of VP1-244E had abolished the heparin-binding of the IEQ variant. To investigate the role of VP1-K244E mutation in heparin-binding and virulence in mice, we introduced this mutation into the IEQ variant through site-directed mutagenesis. The IEQ variant with K244E mutation (termed IEQ-244E) however failed to achieve high virus yield in cell culture for subsequent *in vivo* experiments. We thus collected IEQ-244E and IEE from the brain homogenates (indicated with ^+^) for subsequent experiments, after confirming the sequences using Sanger sequencing. IEQ-244E^+^ displayed significant reduction of heparin-binding compared to clone-derived IEQ and IEE, and IEE^+^ (Fig 3A). The IEE input was generated after one passage in RD cells, and next-generation sequencing (NGS) revealed a mixed population of 98E, 98K and 145E. In contrast, the IEE^+^ from brains and muscles showed dominance of 98E and 145E, which explained the weak heparin binding observed (S1 Table).

To determine the association of heparin-binding and *in vivo* virulence, we then infected one-day old suckling mice with clone-derived IEE and IEQ-244E^+^ by i.p. administration. Brain homogenate from IEQ-infected surviving mice was also harvested and used as a negative control (IEQ^+^; viral RNA not detected in RT-PCR). At day 4 post-infection, 100% mortality was observed in IEE and IEQ-244E^+^-infected mice but none of the mice succumbed to IEQ^+^ infection (Fig 3B).

The hind limb and brain samples from IEE and IEQ-244E^+^-infected mice were then processed for histopathological analysis, and results supported earlier findings. Immunohistochemical (IHC) examination revealed massive localization of viral antigens in both IEE and IEQ-244E^+^-infected muscles, indicating that skeletal muscle is an important replication site (Fig 3C). Inflammation and extensive muscle damage were also observed in the haematoxylin and eosin (H&E)-stained sections of muscle. Mild inflammation was detected in the cortex. Viral antigens were detected in neuron bodies and their axon dendrites mainly distributed in the pons and dentate nucleus (Fig 3D). Mononuclear cell infiltrates were also evident in the cortices. In contrast, no distinctive histopathological change was observed in the mock-infected organ samples.

### High lethality of weak heparin-binding EV-A71 variants correlates with high viremia

**Fig 4:**
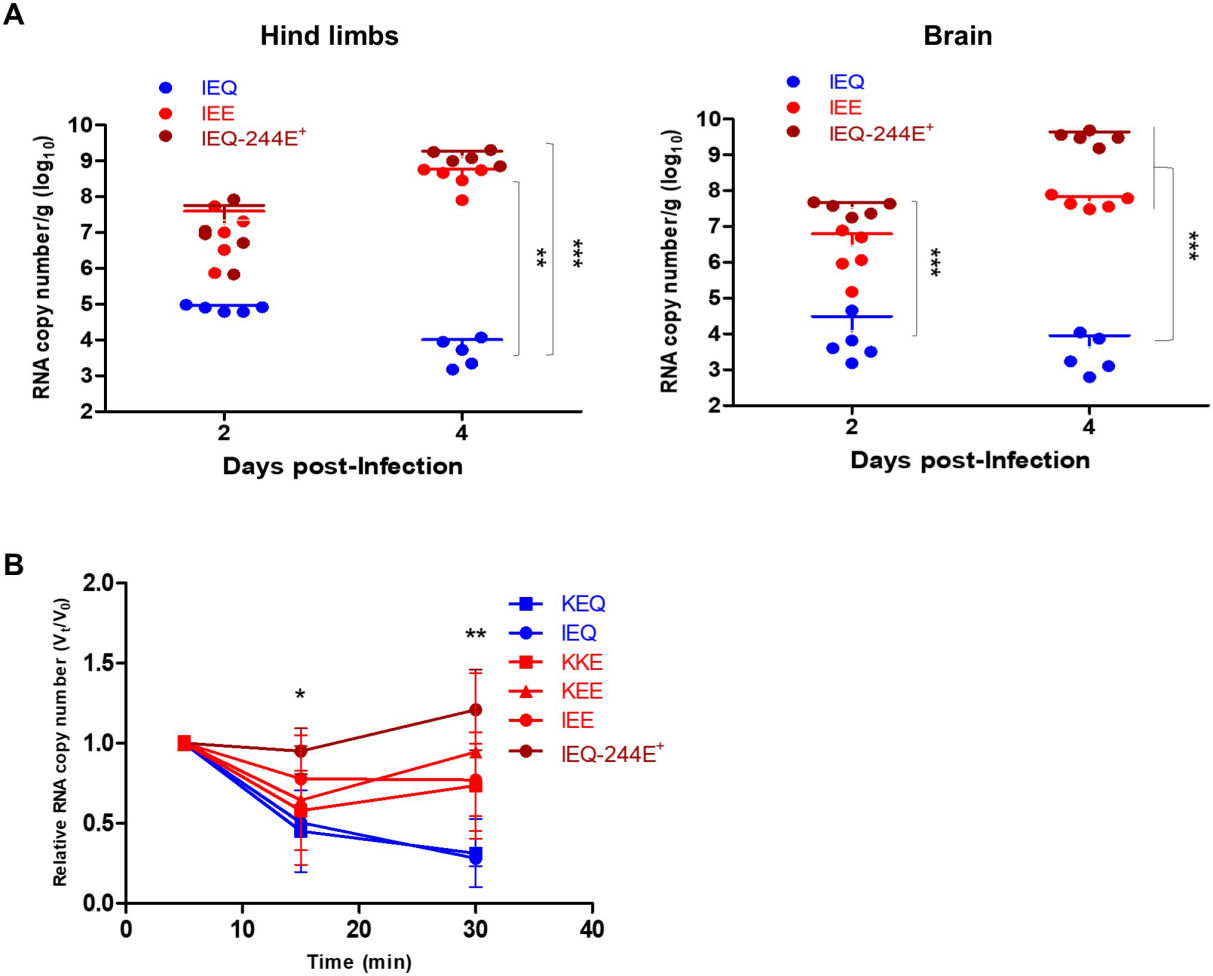
Viral load quantitation from harvested organs and viremia level induced by EV-A71 variants. (A) At selected time points, IEQ, IEE and IEQ-244E^+^-infected mice (n = 5) were sacrificed and viral loads were determined from harvested hind limbs and brains using qRT-PCR. Significant differences between viral variants are labelled as ** (*P* < 0.01) and *** (*P* < 0.001). (B) Virus clearance from blood was quantitated using qRT-PCR following intravenous inoculation of KEQ, KKE, KEE, IEE, IEQ or IEQ-244E^+^ into 3–4 weeks old mice. The viral RNA copies were quantitated at selected timepoints up to 30 minutes. The data are presented as V_t_/V_0_, indicating the fraction of viral RNA copies at each timepoint (V_t_) over the initial viral RNA copies (V_0_). Significant differences between KEQ and IEQ-244E^+^ are labelled as * (*P* < 0.05) and ** (*P* < 0.01).

Strong heparin-binding confers the advantage of promoting virus attachment on the cell surface, thus increasing the probability of virus-functional receptor interaction *in vitro* [9]. However, we have demonstrated that a strong heparin-binding phenotype is deleterious to virus pathogenesis *in vivo*. To unravel the discrepancy of cytopathogenicity *in vitro* and *in vivo* virulence, we investigated virus dissemination in mice. Following i.p. infection, five mice were sacrificed for viral load quantitation in brain and hind limbs. IEE and IEQ-244E^+^ variants replicated to higher titers than IEQ in both hind limbs and brain, at day 2 and 4 post-infection (Fig 4A).

To investigate the association of viremia with *in vivo* pathogenesis, three- to four-week-old mice were infected intravenously with KEQ, KKE, KEE, IEE, IEQ and IEQ-244E^+^ variants. Blood samples were then collected at 5, 15 and 30 min post-inoculation for viral load quantitation. Both strong heparin-binding variants KEQ and IEQ showed rapid viral clearance, with approximately 70% cleared from the bloodstream at 30 minutes (Fig 4B). Weak heparin-binding variants such as KKE, KEE and IEE displayed mild reduction of viremia throughout 30 minutes. Only about 23% of IEE had been cleared by 30 minutes post-infection. A sustained viremia level with minimal clearance was observed in IEQ-244E^+^-infected mice. Our data indicated stronger heparin-binding resulted in low viremia levels in the host, which may be due to adsorption and sequestration of viruses in surrounding tissues. The low viremia level may render the virus less efficient in disseminating to other organs, as seen in KEQ and IEQ variants.

### High fidelity and impaired recombination attenuate *in vivo* virulence of IEQ

**Fig 5:**
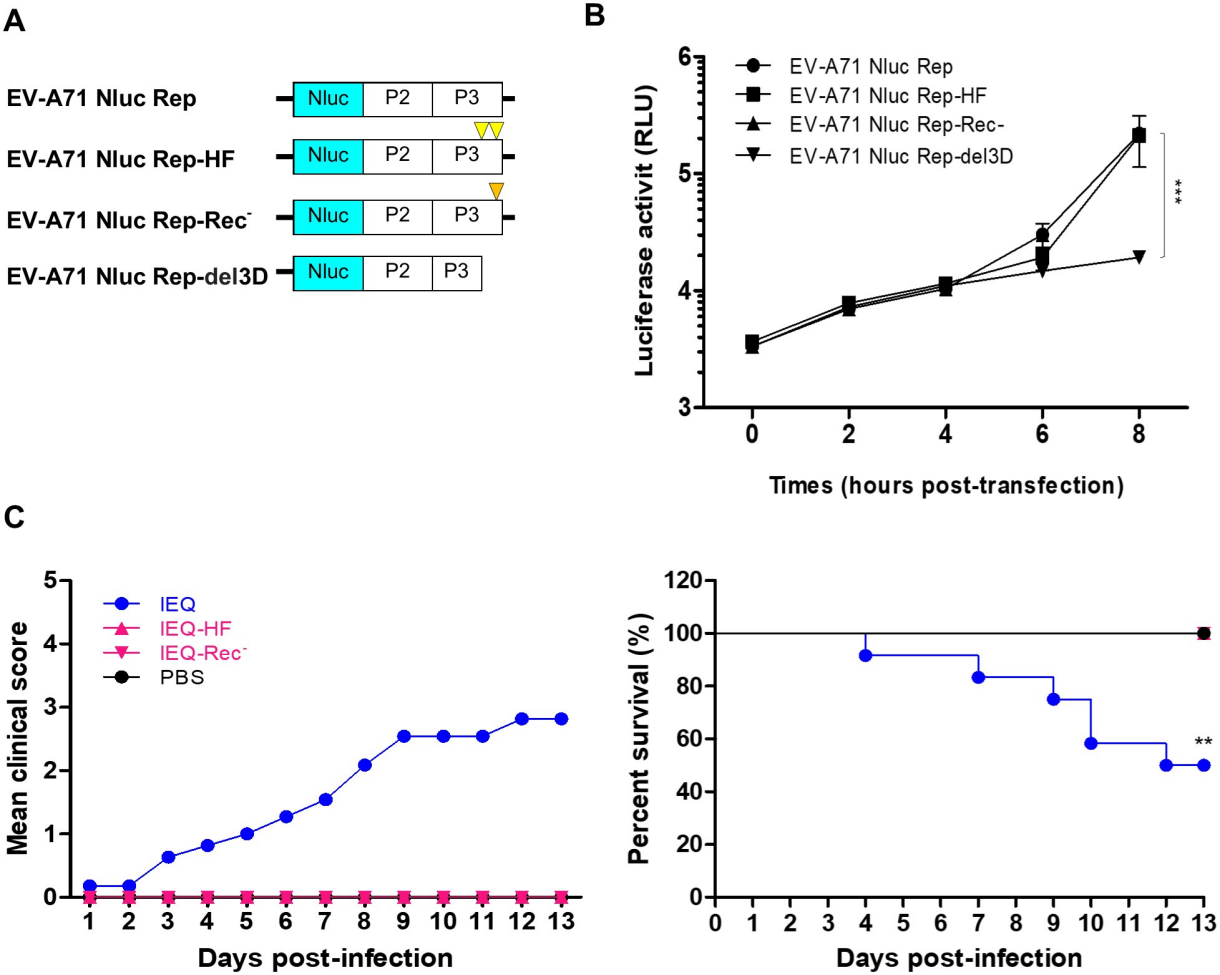
High fidelity and impairing recombination attenuate *in vivo* virulence of IEQ. The RdRp of IEQ was modified to harbor the high fidelity G64R and L123F mutations (HF) or the recombination-deficient Y276H mutation (Rec^-^) and virulence was analyzed in mice. (A) EV-A71 subgenomic replicon (EV-A71 Nluc Rep), EV-A71 Nluc Rep-HF (HF mutations are indicated by yellow triangles), EV-A71 Nluc-Rep-Rec^-^ (Rec^-^ mutation is denoted by orange triangle) and truncated replicon (EV-A71 Nluc Rep-del3D) were transfected separately into RD cells. (B) Luciferase activities were determined up to 8 hours post-transfection. Significant differences between replicon variants and WT are labelled as *** (*P* < 0. 001). (C) Virulence of IEQ-HF, IEQ-Rec^-^ and IEQ following i.p. infection of mice was measured by clinical scores and percentage of survival. IEQ-HF and IEQ-Rec^-^ curves are identical to that of the mock-infected PBS group. Significant difference between viral variants and IEQ is labelled as ** (*P* < 0.01).

The emergence of a weak heparin-binding variant with VP1-244E mutation is the key determinant of *in vivo* adaptation and pathogenesis of IEQ. We hypothesized that IEQ is avirulent without the acquisition of the VP1-244E mutation *in vivo*. To reduce mutation rates and restrict generation of viral quasispecies, we engineered the viral RNA-dependent RNA polymerase (RdRp) of IEQ to harbor previously identified high-fidelity mutations G64R and L123F (abbreviated as HF in Fig 5A) [42–44] and recombination-deficient mutation Y276H (labelled as Rec^-^) [45]. We employed a luciferase-based replicon system to assess the impact of these mutations on genome replication. As demonstrated in Fig 5B, no significant differences in luciferase activities were observed between wild type EV-A71 Nluc Rep, EV-A71 Nluc Rep-HF and EV-A71 Nluc Rep-Rec^-^, suggesting that these mutated RdRp variants replicate as efficiently as the wild type. Next, we generated and rescued the IEQ-HF and IEQ-Rec^-^ virus variants. These IEQ-HF and IEQ-Rec^-^ variants were genetically stable with no reversion of mutations nor emergence of VP1-244E observed after a few passages, in addition to indistinguishable plaque morphology to IEQ (S2 Fig).

To characterize the impact of increased fidelity and recombination deficiency on *in vivo* virulence, one-day old suckling mice were infected with IEQ, IEQ-HF and IEQ-Rec^-^. Half of the IEQ-infected mice died by day 12 post-infection (Fig 5C), while none of the mice died following IEQ-HF and IEQ-Rec^-^ infection. Attempts to recover IEQ-HF and IEQ-Rec^-^ viruses from all the surviving mice were unsuccessful. IEQ-HF and IEQ-Rec^-^ did not result in any infections, and attenuated *in vivo* virulence of IEQ.

### Emergence of VP1-244E is important for neuroinvasion

**Fig 6:**
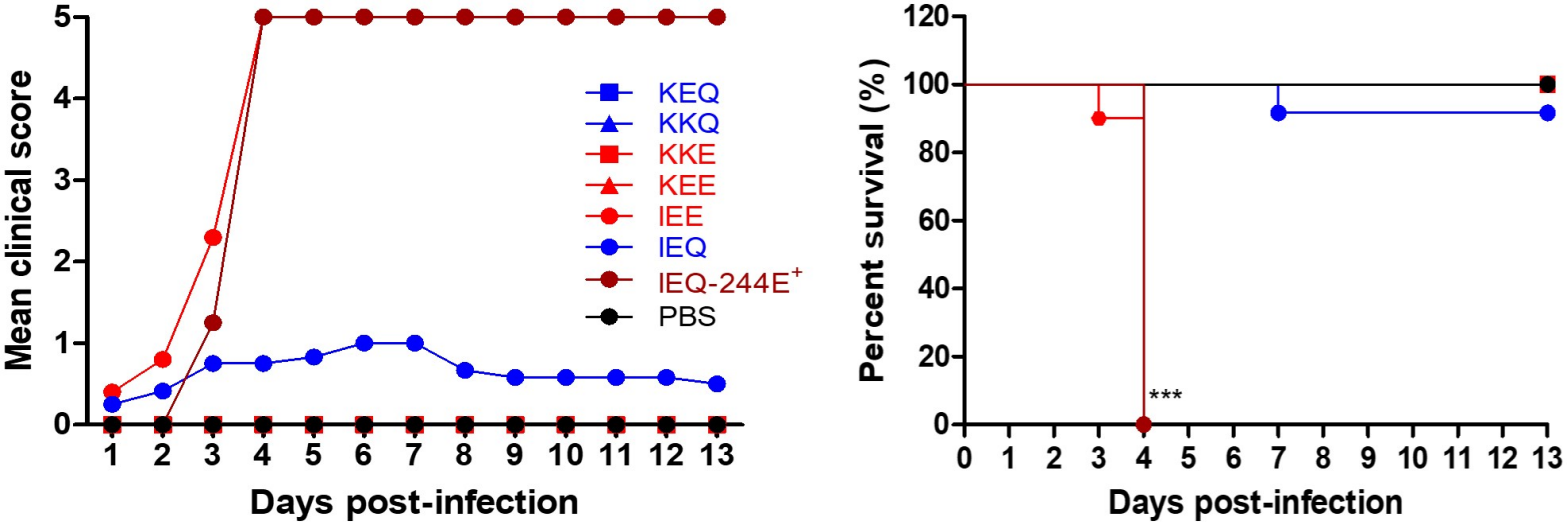
IEQ virulence was attenuated when inoculated intracerebrally. A dose of 1 × 10^5^ PFU of each EV-A71 variant was administered intracerebrally into one-day old suckling mice. Clinical scores and percentage of survival over 13 days post-infection are shown. Significant differences between viral variants and WT are labelled as *** (*P* < 0.001).

To determine if emergence of the VP1-244E mutation is critical for systemic dissemination, we next examined neurovirulence (the ability to directly infect the CNS) of all the EV-A71 variants following direct intracerebral inoculation. A dose of 1 × 10^5^ PFU of each of the EV-A71 variants was intracerebrally injected into separate litters of one-day old mice (Fig 6). Similar mortality rates (100% mortality at day 4 post-infection) were shown by the IEE and IEQ-244E^+^ variants, the former having been earlier shown to be highly lethal following i.p. infection (Fig 2A). Notably, IEQ infection, which caused 66.7% mortality when inoculated intraperitoneally, now showed a drop of virulence to 8.3% (n = 1) mortality following i.c. infection. Detection of the VP1-K244E mutation by sequencing in organ samples from dead IEQ-infected mice further confirmed its importance as a neuroinvasion determinant. No virus was detected in the remaining mice with no disease symptoms. Taken together, our data indicates that the critical mutation conferring neuroinvasive phenotype to the IEQ variant is VP1-244E, which mainly arises during systemic dissemination when IEQ is inoculated intraperitoneally and not directly into the brain.

### Uncharged VP1-244 intermediate variants emerged during transition to IEQ-244E

**Fig 7:**
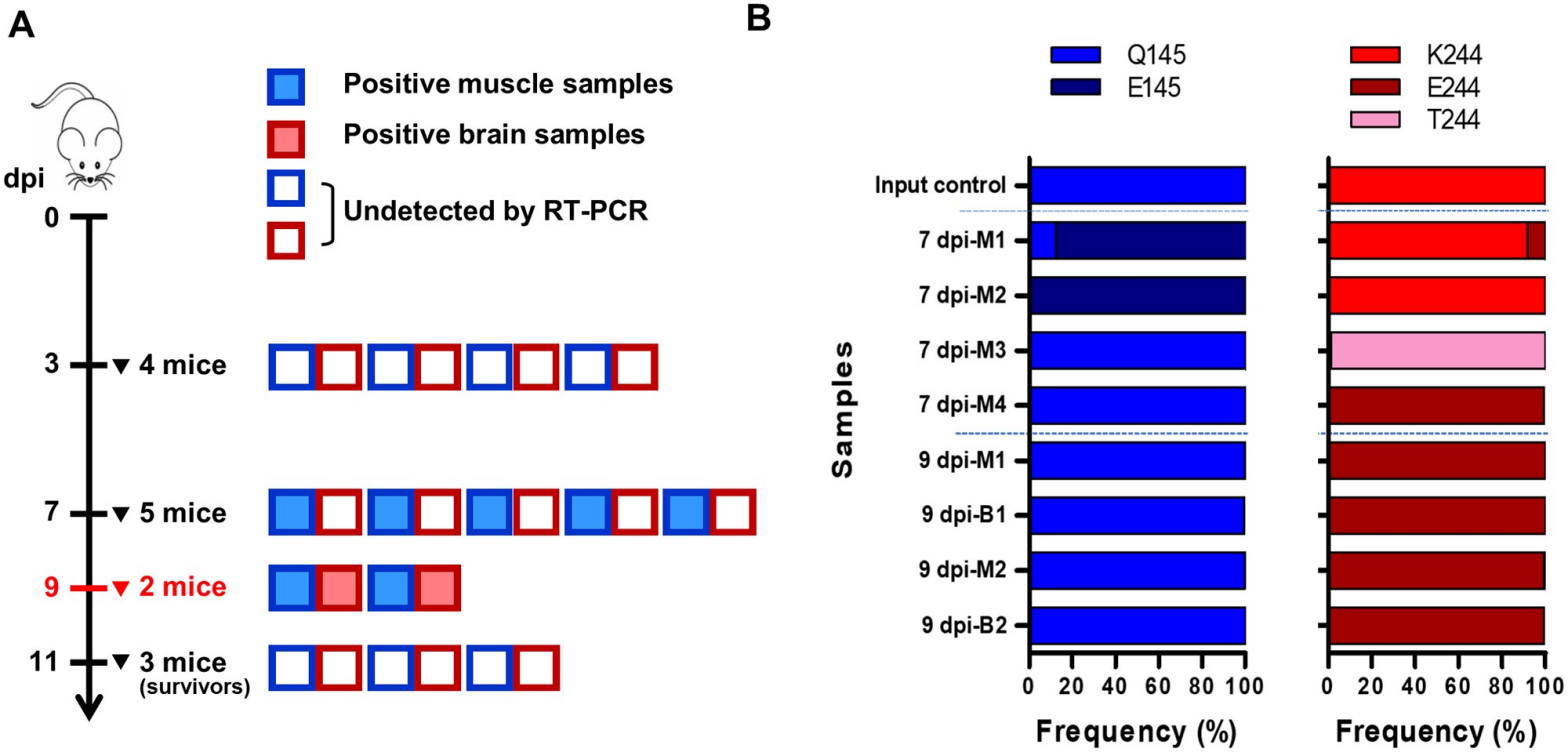
Sequential emergence of K244E mutation involves intermediate transition variants. One-day old suckling mice were intraperitoneally injected with 1 × 10^5^ PFU of IEQ. (A) At days 3 and 7 post-infection, mice were euthanized to harvest their hind limb muscles and brains. At day 9 post-infection, two moribund mice were sacrificed for processing (highlighted in red). At the end of the experiment (day 11 post-infection), the remaining three healthy mice were sacrificed and categorized as ‘survivors’. The boxes indicate RT-PCR results for EV-A71 for each muscle and brain sample. A total of nine samples were positive, comprising five muscle samples from day 7 post-infection, and two muscle and brain samples from day 9 post-infection. (B) NGS results showing the frequency of different variants at the VP1-145 and VP1-244 sites. Note that 7 dpi-M5 sample had poor sequencing coverage. M indicates muscle; B indicates brain.

To investigate how the VP1-K244E mutation emerges during *in vivo* infection, 14 suckling mice were intraperitoneally infected with IEQ. The mice were sacrificed at days 3, 7 and 11 post-infection to harvest hind limbs and brains for NGS of the virus population diversity. At day 9 post-infection, two moribund mice were collected. We first screened all the harvested samples using RT-PCR. At day 3 post-infection, none of the collected organs were positive for EV-A71 (Fig 7A). All five muscle samples collected were positive for EV-A71 at day 7 post-infection, suggesting that viruses were replicating in skeletal muscles. Brain samples were negative suggesting that limited virus had disseminated to the brain at this time point. As expected, both muscle and brain samples collected from the moribund mice at day 9 post-infection were positive for EV-A71. None of the surviving mice collected at day 11 post-infection were positive for EV-A71 RNA in both hind limbs and brain.

Deep sequencing of the EV-A71-positive organ samples revealed changes mainly at position VP1-145 and VP1-244 (Fig 7B). Interestingly, Q145E mutation was detected in hind leg muscle samples 7 dpi-M1 and M2 with frequency of over 80%. We observed a sequential transition of 244K to 244E from day 7 to day 9 post-infection. At day 7 post-infection, VP1-244K was predominant in two out of five hind limb samples (M1 and M2), while only a single sample (M4) showed VP1-244E as the dominant viral population. Notably, we identified a substitution, VP1-K244T from the hind limb of sample 7dpi-M3 with high frequency of over 90%. Sample 7 dpi-M5 also showed 244T and 244E, but due to poor sequencing coverage it was eliminated from the analysis. The emergence of an uncharged 244T intermediate appears to be important during the transition from positively-charged 244K to negatively-charged 244E, which is a radical change in physicochemical properties. As infection progressed, VP1-244E was solely detected in the organs harvested at day 9 post-infection, further affirming the contribution of VP1-244E to *in vivo* virulence. Apart from VP1-145 and 244, we also detected other non-synonymous mutations previously linked to heparin-binding from the harvested samples, including VP1-L97R, N104S and E167G (Table 1).

**Table 1:** Non-synonymous mutations related to heparin binding detected from different organ samples of IEQ-infected mice.

Samples		97L		98E		104N		145Q		167E		244K	
		Mutation	Frequency (%)	Mutation	Frequency (%)	Mutation	Frequency (%)	Mutation	Frequency (%)	Mutation	Frequency (%)	Mutation	Frequency (%)
**Input control (IEQ)**		-	-	E98Q	5.70	N104T	2.51	-	-	E167G	19.12	-	-
		-	-	E98G	3.71	N104S	8.96	-	-	E167Q	3.62	-	-
		-	-	E98V	1.86	-	-	-	-	-	-	-	-
**7 dpi**	**M1**	-	-	-	-	N104S	90.32	Q145E	88.58	E167G	8.62	K244E	8.21
**7 dpi**	**M2**	-	-	-	-	-	-	Q145E	99.72	E167G	99.68	-	-
**7 dpi**	**M3**	-	-	-	-	N104S	99.28	-	-	-	-	K244T	98.67
**7 dpi**	**M4**	-	-	-	-	N104S	98.12	-	-	-	-	K244E	99.95
**9 dpi**	**B1**	-	-	-	-	N104S	99.80	-	-	-	-	K244E	99.71
**9 dpi**	**M1**	-	-	-	-	N104S	99.39	-	-	-	-	K244E	99.75
**9 dpi**	**B2**	L97R	47.99	-	-	-	-	-	-	E167G	50.96	K244E	99.38
**9 dpi**	**M2**	L97R	99.40	-	-	-	-	-	-	-	-	K244E	99.59

Note: -indicates no variation >1% was observed. M indicates muscle, B indicates brain.

To test if VP1-244E variant will develop specific mutation or reversion during cell culture adaptation, the IEQ-244E^+^ homogenates harvested from muscle and brain were propagated in RD cells. No significant CPE was observed in passage 1, and therefore the cell supernatants were collected for further propagation. At passage 2, mild CPE was observed with three out of four samples showing VP1-244E had reverted very quickly to 244K (S3 Fig). Clearly, reversion of E244K frequently emerged during *in vitro* culture adaptation.

### Weak heparin-binding is due to loss of electrostatic interactions at the five-fold axis

**Fig 8:**
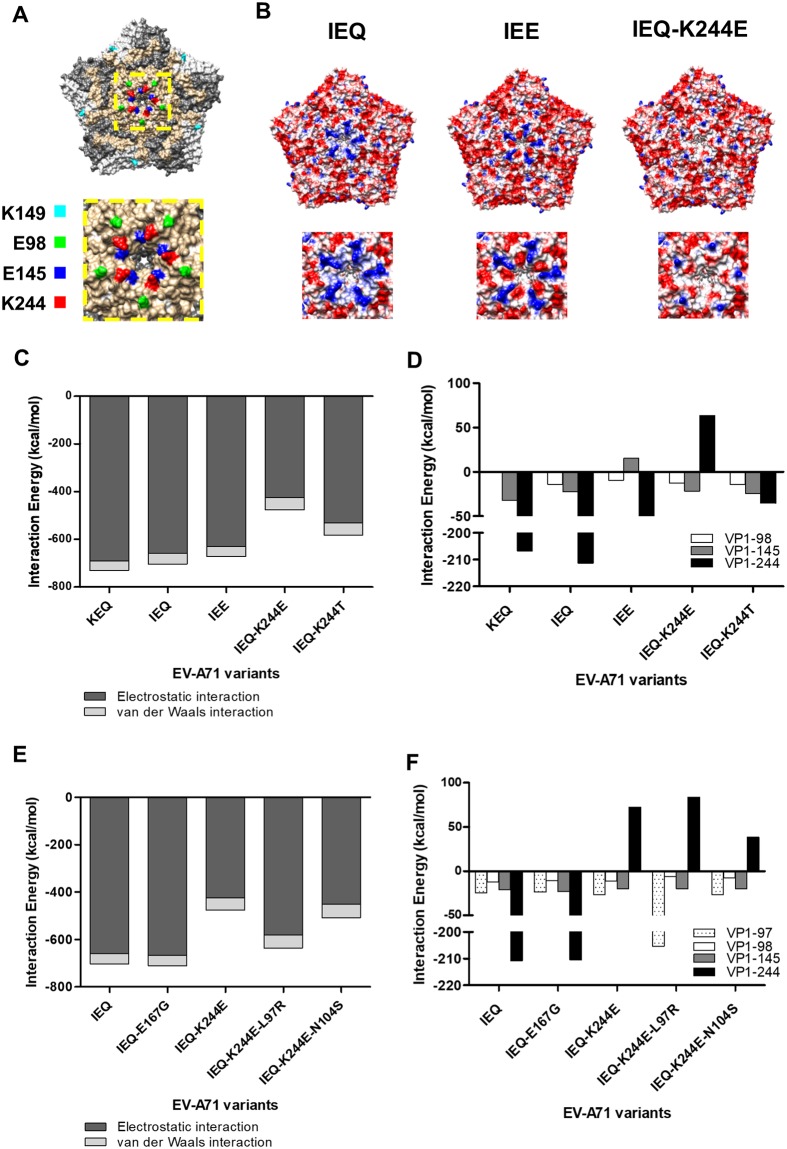
Comparison of electrostatic surface properties of the EV-A71 structure. The analyzed amino acid residues are labelled in different colors in the EV-A71 capsid pentamer structure (PDB ID: 4AED). (A) The front views of the EV-A71 pentamer are displayed along with a magnified view of the five-fold axis using Chimera 1.10.1. (B) Electrostatic surface properties of IEQ, IEE and IEQ-244E variants were examined. Electrostatic charges are contoured from red (-5 kcal/mol·e, negatively-charged) to blue (+5 kcal/mol·e, positively-charged). Magnified views of the five-fold axis are shown for each variant. Total interaction energy (C) and individual interaction energy of residues (D) within a 4Å radius of EV-A71 variants and VP1-244 variants docked to heparin were evaluated. Total interaction energy (E) and individual interaction energy of residues (F) within a 4Å radius of other mutations detected from NGS docked to heparin were examined.

IEE was experimentally proven to be highly lethal in mice. However, we observed that IEQ selectively acquires VP1-K244E over the VP1-Q145E mutation to gain neuroinvasiveness and neurovirulence (Figs 3B & 6). We reasoned that VP1-244E exhibits weaker heparin-binding ability compared to VP1-145E, and therefore, could be favorably selected *in vivo*. We employed *in silico* analysis to characterize the heparin-binding affinity of VP1-145E and VP1-244E. VP1-98, 145 and 244 are located around the five-fold axis of the EV-A71 pentamer (Fig 8A). Based on the electrostatic maps, the five-fold axis of the IEQ variant is highly positive-charged (Fig 8B), implying strong affinity to heparin. With the VP1-Q145E substitution, IEE has lower positive charges at its five-fold axis. Since the VP1-244K residue is protruding from the surface of the five-fold axis, substitution of a positively-charged lysine residue to a negatively-charged glutamic acid at this position greatly reduces the electrostatic potential on the five-fold axis and could change the capsid conformation.

Simulated docking of 12-mer heparin to EV-A71 residues within a 4Å radius revealed no notable change in interaction energies between KEQ (-730.76 kcal/mol) and IEQ (-703.71 kcal/mol) (Fig 8C). The VP1-Q145E mutation of IEE resulted in a drop of 32.34 kcal/mol interaction energy when compared to KEQ. The IEQ-244E variant showed the weakest heparin-binding ability as the interaction energy drastically dropped to -476.32 kcal/mol. Compared to the IEQ-244E variant, the uncharged IEQ-244T variant exhibited higher interaction energies of -552.21 kcal/mol and -582.89 kcal/mol, respectively. The energy change was mainly contributed by the VP1-244 residue, with a descending energy order of 244K, 244T and 244E, which correlates with the scale of strong to weak heparin-binding (Fig 8D).

When examining the heparin docking of non-synonymous variants detected by NGS, the additional VP1-E167G mutation had no effect on interaction energy compared to IEQ (Fig 8E). Interestingly, variants with VP1-L97R and VP1-N104S mutations showed slight increase in the interaction energy of IEQ-244E variants, suggesting an enhanced effect of heparin-binding. Although IEQ-244E-97R showed very weak binding strength similar to IEQ-244E, a compensatory effect was seen in the 97R site which singly contributes to a more negative interaction energy, i.e. strong heparin-binding (Fig 8F). Meanwhile, the additional VP1-N104S mutation has also increased the heparin-binding of IEQ-244E variant. Taken together, different compensatory mutations could emerge to overcome the capsid instability and alter the virus fitness.

## Discussion

As heparin-binding phenotype has been implicated in virulence of some neurotropic viruses, we studied the relationship between heparin-binding phenotype and mouse neurovirulence in EV-A71. Our data highlighted the key role of electrostatic interactions in shaping heparin-binding to confer virulence in mice. Among the weak heparin-binding variants used in this study (KKE, KEE and IEE), only IEE was associated with increased virulence and virus fitness *in vivo*. Strikingly, IEQ, which should be a strong heparin binder, showed high virulence, and we showed that this was due to the mutation VP1-244E, which conferred weak heparin-binding. This VP1-244E mutation has been previously identified as a mouse virulence determinant [46, 47]. Increasing polymerase fidelity or impairing recombination of IEQ abolishes *in vivo* neurovirulence possibly by restricting the emergence of VP1-244E, suggesting the importance of viral population diversity in EV-A71 pathogenesis, as reported for poliovirus [48, 49]. We showed that selection of adaptive mutations around the five-fold axis with roles in heparin-binding impacts viral pathogenicity.

Six EV-A71 variants were engineered with different amino acids at VP2-149, VP1-98 and VP1-145. VP1-98 and VP1-145 also have important roles in binding to PSGL-1 found in blood cells [8]. Both VP1-145 and VP2-149 have been implicated in mouse adaptation and virulence [24, 25, 27, 30, 46]. The VP1-98 and VP1-145 residues also act as modulators of heparin-binding in cell culture [39]. Heparin-binding may impact virulence outcome through neuroinvasion or neurovirulence [50]. Unlike SCARB2, heparin has not been showed to have roles in EV-A71 viral uncoating and internalization [7, 51–53], suggesting that heparin-dependent virulence may not to be due to direct virus-functional receptor interaction. In mice, mSCARB2 and mPSGL-1 are known to poorly support EV-A71 infection [51, 54, 55]. Therefore, an unidentified mouse receptor could be utilized to achieve high viremia and dissemination. Many reports have shown that the mutations in our engineered variants could result in better binding to mouse cells after adaptation [25, 56, 57]. As different cells may have different expression of EV-A71 receptors, tissue tropism and receptor availability will warrant further investigations. Also, KKE a poor heparin-binder, is avirulent *in vivo*. Heparin-binding is not solely controlled by a single mutation, and compensatory or complementary mutations may change weak heparin binders to strong binders. Other unknown receptors and additional mechanisms may also be involved in *in vivo* virulence.

Establishment of viremia is crucial for further dissemination to other target tissues such as skin and invasion into CNS [58]. During *in vivo* dissemination, the strong binding affinities of heparin binders KEQ and IEQ increase the likelihood of virus being sequestered by tissue GAG, resulting in rapid virus clearance from blood circulation [34, 36, 59]. This gives rise to a low viremia level with a substantial reduction of virulence in mice. Kobayashi and colleagues reported that KEG virus is less virulent compared to KEE due to the former harbouring the VP1-145G residue, enabling the virus to adsorb more strongly to heparin, resulting in attenuated virulence in SCARB2-expressing transgenic mice [60]. Similarly, IEE, KEE and KKE were cleared slowly in the bloodstream, and the poor heparin-binder IEQ-244E^+^ remained in the blood. Our findings that a strong heparin-binding phenotype attenuates virulence is also observed in other viruses, including Sindbis virus [36], Venezuelan equine encephalitis virus [59], West Nile virus [33], yellow fever virus [34] and Japanese encephalitis virus [61]. Using a monkey model, Zhang *et al*. showed that the establishment of viremia was strongly correlated with EV-A71 neuroinvasion into CNS [16]. A clinical study correlating prolonged viremia in EV-A71 patients with severe CNS involvement further suggests the importance of viremia in determining severity outcome [62].

We performed i.c. infection to bypass peripheral dissemination and neuroinvasion, therefore directly measuring the neurovirulence of each variant. Unlike the weak heparin binders IEE and IEQ-244E^+^, intracerebrally inoculated IEQ failed to exhibit the same neurovirulent phenotype as it did following i.p. administration, for which we propose two possible explanations. First, virus replication in brain cells may be restricted. This is supported by histopathological studies showing very few neurons in brain are infected with EV-A71 [63, 64]. IEQ viruses failed to acquire the VP1-K244E mutation, presumably due to lower infectivity of neurons and suboptimal replication following direct inoculation into brain, resulting in low virulence in mice. Secondly, viral multiplication in extraneural tissues such as hind limb skeletal muscle plays a key role in neuropathogenesis [65]. Mice intracerebrally inoculated with IEE showed higher viral load in hind limb muscles (S4 Fig), implying that the virus spread to peripheral tissues and underwent further extraneural replication (especially in the skeletal muscles) before re-entering the brain at high titers [16, 27, 65]. Retrograde axonal transport is the main transmission route for neuroinvasion [17, 64]. High replication will result in muscle damage that increases retrograde axonal transport and virus trafficking to the CNS [17, 66, 67]. The weak heparin binders IEE and IEQ-244E^+^ remain lethal since they disseminate effectively and establish high viremia prior to neuroinvasion. Using an *in vitro* porcine blood brain barrier (BBB) model (S1 Text), we found no correlation between heparin-binding and neuroinvasion across the BBB through tight junction leakages, as demonstrated by IEE and IEQ which induce poorer permeability compared to wild type KEQ (S5 Fig). This suggests that hematogenous spread is not the main route of spread to brain [68]; rather, establishment of high viremia appears crucial for virus dissemination to other target organs which support high levels of replication.

The mechanism of pathogenesis associated with heparin-binding is driven by electrostatic interactions at the five-fold axis of the virus surface. Alteration of the five-fold axis may affect capsid instability resulting in conformational changes which trigger genome uncoating, bypassing the need for receptor-virus binding. Mouse adaptation in poliovirus is controlled by a balance between capsid plasticity during uncoating and thermostability of the virion [69]. Similarly, electrostatic repulsion around the five-fold axis which results in capsid instability is observed in naturally thermo-labile foot-and-mouth-disease virus [70]. Dynamic switching between weak and strong heparin-binding phenotypes has been observed *in vitro* [39, 71]. In IEQ, VP1-145E was detected in samples harvested at early timepoints (7 dpi) but was 145Q at later timepoints (9 dpi). Multiple VP1-244 variants (244K, 244T and 244E) were generated with different effects on electrostatic interactions with heparin (K>T>E). As K to E represents a non-conservative substitution, the virus has evolved to transition through a non-charged residue (T) which has lower fitness cost during viral dissemination [72–74]. As GAGs are ubiquitous in tissues, a natural selective pressure thus exists to revert weak heparin-binding variants to heparin-binding variants which can then attach to and infect a wide range of tissues. This observed dynamic switching between weak and strong heparin-binding phenotypes highlights the importance for the virus to maintain an optimal electrostatic interaction for stable capsid conformation.

Three complementary mutations, VP1-L97R, VP1-N104S and VP1-E167G, were detected along with VP1-K244E from IEQ-infected moribund mice. Given that these mutations are near the VP1-244 residue site and located at loops (S6A Fig), they could be selectively utilized by the virus to stabilize the conformational structure of the VP1-K244E mutation at the five-fold axis. The VP1-N104S mutation was frequently associated with the VP1-244 variants. Root mean square fluctuation (RMSF) was measured to examine the dynamic movement of residues (S6B Fig); the RMSF value of the BC loop (where VP1-104 is located) was relatively higher in IEQ-244E compared to other virus variants. This indicates that residues within the VP1 BC loop are flexible and may bind poorly to heparin. The emergence of the VP1-N104S mutation could contribute to the conformational stability of VP1-K244E, resulting in enhanced viral infectivity [75]. The VP1-L97R mutation, within the VP1 BC loop, has been reported to enhance heparin-binding [76]. This mutation was first detected from EV-A71 in the blood, CSF and stool from an immunocompromised patient [77]. However, we found this mutation in both the skeletal muscle and brain of an IEQ-infected moribund mouse. Similarly, the presence of the VP1-L97R could stabilize the conformational structure of VP1-244E. Interestingly, both VP1-97R and 104S did not co-exist in the same sample. The VP1-D31G [78] and VP1-E167G [77] mutations previously implicated in neurotropism in humans were also observed in the brain samples but at very low frequencies. Furthermore, restricting polymerase fidelity and impairing recombination rendered the virus avirulent, suggesting that restriction of population diversity alters fitness of the virus *in vivo*. The transition of different virus subpopulations is pivotal for the virus to gain fitness and adaptation *in vivo*.

We speculate that our findings are not only limited to the murine model but may reflect neuropathogenesis in humans. Strong heparin-binding variants (KEQ and EGK) have been more frequently detected from sequencing of virus cultures than from direct sequencing of clinical specimens, suggesting that heparin-binding phenotypes are a consequence of adaptation to cell culture (S2 Table, S1 Appendix). Detection of VP1-145E in the sequences of a fatal encephalitis autopsy specimen [79] further reinforces our view that weak heparin-binding is associated with virulence in humans, as we have shown in mice in the present study. EV-A71 infection is usually mild and limited to HFMD, with neurological complications seen in 0.1–1.1% and deaths in 0.01% to 0.03% [4, 80–83]. The frequent reversion between heparin-binding and weak heparin-binding variants fulfils the trade-off hypothesis in which the virus juggles between the virulence and transmission. IEE and IEQ-244E are completely lethal whereas the IEQ variant demonstrated intermediate virulence in mice.

**Fig 9:**
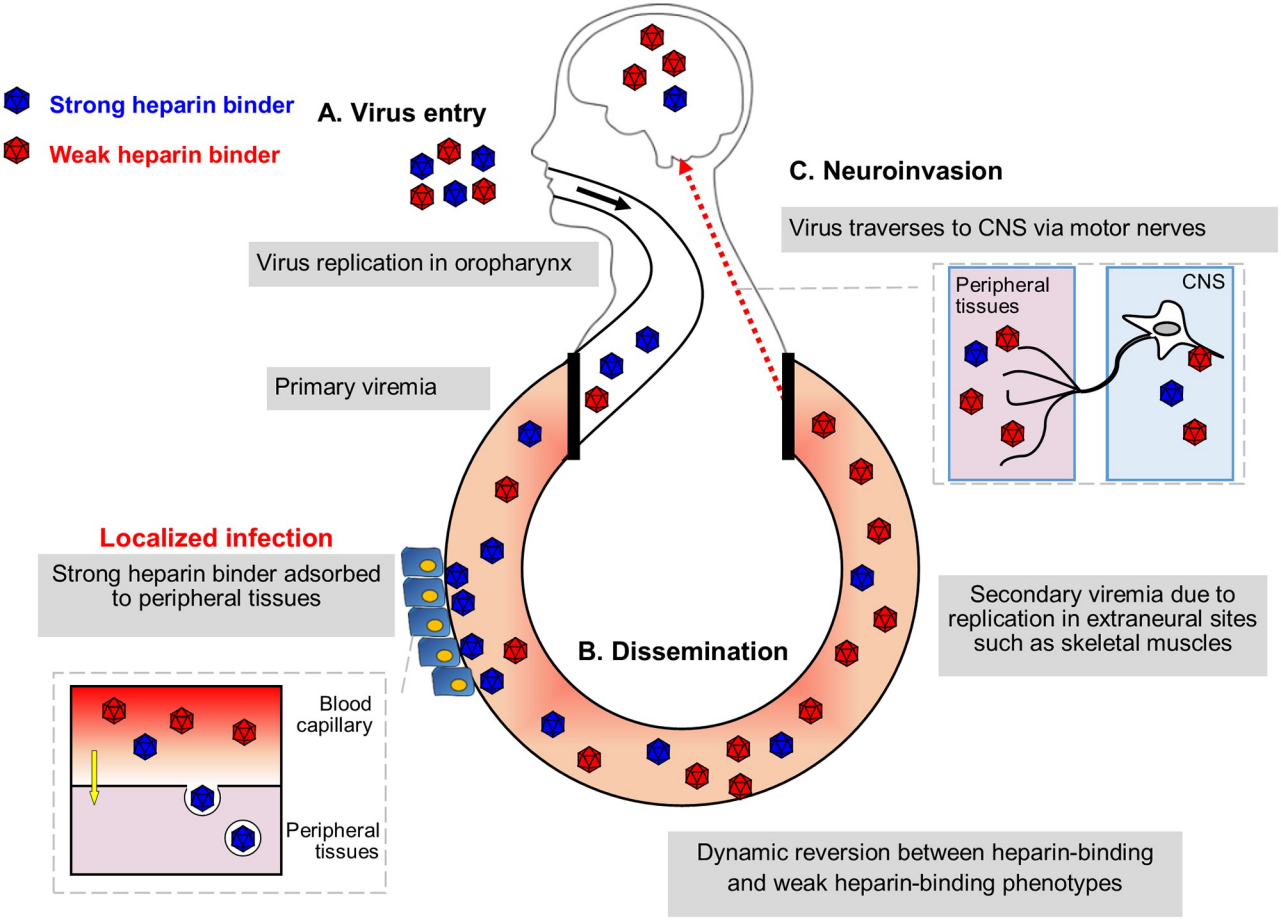
Hypothesized model of EV-A71 heparin-dependent pathogenesis in human. Three major factors are responsible for EV-A71 virulence determination, namely virus entry, peripheral dissemination and neuroinvasion. (A) Both strong and weak heparin binders infect humans at the same rate, using the same inoculation route and receptor. (B) Viremia is established upon virus entry. Strong heparin binders are more readily removed from the blood circulation by binding to peripheral tissues due to their high affinity to heparin. Meanwhile, weak heparin binders give rise to higher viremia with better dissemination to other organs. (C) Neuroinvasion occurs when virus travels from peripheral motor nerves to the CNS via retrograde axonal transport.

We proposed a hypothetical EV-A71 pathogenesis model to show the importance of heparin-binding in human infection (Fig 9). Three determinant factors of EV-A71 virulence are virus entry, dissemination and neuroinvasion. Both strong and weak heparin binders could infect humans, probably at different susceptibility. Primary viremia is established upon virus entry in primary replication sites such as tonsils and oropharynx [15]. Strong heparin binders are absorbed to GAG-rich tissues and readily removed from blood circulation due to their high affinity to heparin, thereby reducing the viremia level. Weak heparin binders are not adsorbed into tissues, but remain in the bloodstream, undergoing further extraneural replication in skeletal muscles, giving rise to high viremia. Upon overcoming the immune system, the high viremia results in better replication and dissemination to skeletal muscles, and peripheral motor nerves, through which the virus invades the CNS by retrograde axonal transport [15, 68]. High viremia alone does not result in direct CNS invasion as the virus cannot traverse the BBB. Many research questions however remain. What additional host factors could alter the heparin-binding phenotypes? Could the host immune responses influence the fate of the viruses if activated early enough before the high viremia stage?

Our current results have several limitations. We used i.p. and i.c. routes, and both are not the natural route of infection in humans. It would be interesting to use the oral route and examine the virulence in mice in future. The animal models used here were 1-day old suckling mice lacking of mature immune system, and both hSCARB2 and hPSGL-1 receptors. The use of hybrid mouse models such as hSCARB2/mSCARB2, hSCARB2 with *stat-1* (interferon signalling) knock-out can provide efficient virus replication and mimics human pathogenesis [84, 85]. The use of original clinical isolates without cell culture propagation for *in vivo* infection may help to mimic infection in human. Also in the study, only VP1 was sequenced, and there may be other genomic changes within the virus genome.

In summary, we showed that weak heparin-binding EV-A71 is highly virulent in mice, in contrast with strong heparin binders which show higher replication *in vitro* due to culture adaptation. This study shows that weak heparin-binding EV-A71 is preferentially selected to disseminate via the bloodstream; in contrast, strong heparin-binding EV-A71 is adsorbed to peripheral tissues and rapidly cleared. The electrostatic surface charges at the VP1 capsid shape heparin-binding and hence EV-A71 virulence. Our findings provide the mechanistic action of heparin-dependent virulence, and have potential therapeutic implications for viruses which utilize heparin as an attachment receptor and are dependent on high viremia levels to cause infection.

## Materials and methods

### Ethics statement

The animal experiments were carried out in accordance with the rules and guidelines of the Animal Experimental Unit (AEU) in University of Malaya. The protocols were reviewed and approved by the Institutional Animal Care and Use Committee of the Faculty of Medicine, University of Malaya (reference number: 2016-190908/R/TCW).

### Cell lines and viruses

Human rhabdomyosarcoma (RD, ATCC no.: CCL-136) was propagated in Dulbecco’s Modified Eagle’s Medium (DMEM) (Life Technologies). All cells were supplemented with 10% fetal bovine serum (FBS). Infected cells were maintained in media containing 2% FBS. All cells were maintained at 37°C in 5% CO_2_.

EV-A71 strain 41 (5865/SIN/000009, GenBank accession no. AF316321; subgenogroup B4) was used for construction of infectious clones using a DNA-launched strategy as reported previously [86]. Unlike its original sequence, this lab strain had been propagated in tissue culture previously and had acquired a VP1-E145Q mutation. We have denoted this strain as KEQ.

Different mutations were incorporated into the EV-A71 infectious clone plasmid using Q5 high-fidelity DNA polymerase (NEB) PCR site-directed mutagenesis with primers listed in S3 Table. The purified PCR products were treated with T4 polynucleotide kinase, T4 ligase and *Dpn*I (NEB) for 1 hour at room temperature. The ligation mixture was then transformed into *E*. *coli* XL-10 GOLD ultracompetent cells (Agilent Technologies). The plasmids were transfected into RD cells using Lipofectamine LTX (life Technologies) as reported previously [39]. The transfected virus stock (P0) was further propagated in RD to generate P1 stock for all experiments.

### Binding of EV-A71 particles to immobilized heparin sepharose beads

A binding assay was performed using columns with immobilized heparin sepharose beads as previously reported [39]. In brief, 200 μl of heparin sepharose (Abcam, UK) was aliquoted into a Pierce Spin Cup with cellulose acetate filter (Thermo Scientific, USA). The heparin sepharose beads were washed twice with binding buffer (0.02 M Tris-HCl, 0.14 M NaCl, pH 7.4), before addition of each virus variant (1 × 10^5^ PFU in 600 μl). The columns containing viruses were incubated for 30 minutes at 4°C, and this was followed by centrifugation and 5 washing steps. The heparin-bound viruses were collected after eluted with elution buffer (0.02 M Tris-HCl, 2M NaCl, pH 7.4). Both virus input and output fractions were quantitated using real-time PCR, and the heparin binding efficiency was normalized by dividing the output viral RNA copy number over the input viral RNA copy number.

### Evaluation of inhibitory effect of soluble heparin on EV-A71 variants

To determine the inhibitory effect of soluble heparin on EV-A71 variants, a virus inactivation assay was performed as previously described [9]. In brief, viruses were incubated with 2.5 mg/ml of soluble heparin (Sigma, USA) for an hour at 37°C. The treated viruses were inoculated onto pre-seeded RD cells and incubated at 37°C. Two days later, the cell viability of each infected virus variants was measured using CellTiter 96 Aqueous One solution Cell Proliferation Assay (Promega, USA). The relative cell viability was calculated with the following formula:

$$\text{Relative}\;\text{cell}\;\text{viability}=\frac{\text{Absorbance}\;\text{of}\;\text{well}\;\text{inoculated}\;\text{with}\;\text{treated}\;\text{virus}\;\text{sample}}{\text{Absorbance}\;\text{of}\;\text{well}\;\text{inoculated}\;\text{with}\;\text{untreated}\;\text{virus}\;\text{sample}}$$

### Mice infection experiments

Groups of one-day old ICR suckling mice (n = 9 to 12) were obtained from AEU. Each group of suckling mice were either intraperitoneally or intracerebrally inoculated with 1 × 10^5^ PFU of each EV-A71 variant or PBS alone. All infected mice were monitored daily for weight change and health status up to 13 days post-infection. A clinical score was recorded using the following grades: 0, healthy; 1, weak or less active; 2, hunched posture and lethargy; 3, one-limb paralysis; 4, two-limb paralysis; 5, moribund or dead. Moribund mice were sacrificed and removed along with any mice found dead. Harvested mice organs were homogenized using hard tissue homogenizing mix (Omni International, USA). RNA was extracted from the homogenates with QIAamp viral RNA mini kit (Qiagen, Germany). The viral loads in organs were determined using TaqMan fast virus 1-step master mix (ABI, USA). One step RT-PCR was also performed to amplify viral RNA from the organs using MyTaq One-Step RT-PCR kit (Bioline, UK) for Sanger sequencing or deep sequencing. Illumina Miseq (Illumina, USA) was performed with 150 nucleotides and 250 nucleotides paired end reads. All listed mutations had an average coverage of at least 20,000 reads unless otherwise stated. The NGS reads were analyzed using CLC Bio Genomic Workbench (Qiagen) and Geneious Prime (Biomatters Ltd, New Zealand). Only variants with frequency >1% were reported.

For the virus clearance assay, 3- to 4 weeks old ICR mice (weighing within 25-35g) were i.p. injected with ketamine/xylazine cocktail prior to infection. Anesthetized animals were then intravenously inoculated with 5 × 10^5^ PFU of each EV-A71 variant via the tail vein. At certain timepoints, blood was collected from anaesthetized mice through the retro-orbital plexus with the use of a sodium heparinized hematocrit capillary (Hirsschmann, Germany). The collected whole blood was then used for viral RNA quantitation.

### Immunohistochemistry

Immunohistochemistry (IHC) were performed by the standard ENVISION technique as described previously [87]. Briefly, deparaffinised and rehydrated tissue sections were blocked using standard immunoperoxidase procedure before antigen retrieval (30 minutes, 99°C, Tris EDTA buffer with 0.05% Tween-20). Tissues were then incubated with rabbit polyclonal EV-A71 VP1 (GeneTex, USA) at 4°C overnight. After washing, tissues were then incubated with goat-anti rabbit HRP-conjugate (Dako, Denmark) for 30 minutes at room temperature. Tissues were stained using DAB (Dako) and counterstained with hematoxylin (Dako). The tissues were mounted using DPX (Dako) prior to examination under a light microscope. The negative control tissues for IHC included mock-infected ICR mice brains (n = 2) and hind-limb muscle tissues (n = 2). Isotype control antibodies or normal rabbit immunoglobulin fractions (Dako) were also used to exclude non-specific staining.

### Electrostatic surface charge analysis of EV-A71 structure

The EV-A71 structure was visualized using Chimera software (UCSF Chimera version 1.10.1, USA). Electrostatic surface potentials of virus capsid were analyzed using the ‘Coulombic surface coloring’ function in which the capsid residues were labelled with different colors based on their electrostatic charges. Positively-charged residues were colored blue while negatively-charged residues were colored red.

### Molecular docking simulation of EV-A71 VP1 and 12-mer heparin

Molecular docking simulation of EV-A71 crystal structure (PDB ID: 4AED) was performed using CDOCKER (CHARMm-based DOCKER) [88], as previously described [39].

### Statistics

All experiments were performed with at least two biological duplicates. Data are shown with error bars indicating standard deviations. Student’s *t*-test was performed for all *in vitro* experiments as well as viral load quantitation from mice organs. Survival of mice was evaluated using Kaplan-Meier analysis. GraphPad Prism version 5.03 (GraphPad Software, USA) was used for statistical analyses with a *P* value of < 0.05 indicating significance.

## Supporting information

S1 Fig EV-A71 variants were confirmed by Sanger sequencing. (TIF)

S2 Fig Comparison of plaque morphologies for IEQ, IEQ-HF and IEQ Rec^-^. (TIF)

S3 Fig Reversion of IEQ-244E^+^ variant sequences after propagation in RD cells. A sequential reversion of VP1-244E to 244K was observed in IEQ-244E+ after cell culture propagation. Note that the 244T variant was also present in the mixed population. The mixed populations of K244 (red), T244 (pink) and E244 (maroon) are shown in different proportions. Only variants with frequency >1% are reported. Note that samples B1 at P0 and B2 at P0 had poor sequencing coverages (between 110–5100). M indicates muscle, B indicates brain.
(TIF)

S4 Fig Comparison of viral loads from muscles and brain in mice infected through the i.c. route. One-day old suckling mice (n = 3) were infected with IEE through i.c. route of administration. At day 4 post-infection, muscles and brains were harvested and viral loads were quantitated using qRT-PCR. Significant comparisons are labelled *** (*P* < 0.001).
(TIF)

S5 Fig Effects of EV-A71 exposure on a porcine *in vitro* BBB model. Illustration of the porcine *in vitro* BBB model which simulates the movement of virus particles through an *in vivo* BBB, in which the luminal side represents the blood capillary while the abluminal compartment represents the brain (A). The *in vitro* model was exposed to different EV-A71 variants with titer of 1 × 10^5^ PFU. The BBB permeability induced by EV-A71 variants were assessed in terms of transendothelial electrical resistance (TEER), with a greater reduction of TEER indicating greater permeability of BBB through tight junction leakages. The TEER was recorded at 2 hours (B) and 6 hours post-exposure (C) along with non-infected cell controls (white bars) and normalized with TEER values measured before virus exposure. Results are presented as mean ± SD (n = 6). Significant differences between viral variants and WT (black bars) are labelled as * (*P* < 0.05) and **(*P* < 0.01), using the Student’s *t* test.
(TIF)

S6 Fig Structural modelling of IEQ-244E variant and root mean square fluctuation analysis of different EV-A71 variants. (A) Structural modelling of VP1 amino acid residues of IEQ-244E (left panel). Each important amino acid is labelled with different colors: VP1-244E in red, VP1-244K in green, VP1-97L in orange and VP1-104N in magenta. Note that VP1-145E is not visible from this angle. The surface of IEQ-244E (right panel) is displayed corresponding to the structural model. (B) Root mean square fluctuation (RMSF) value of VP1 and VP2 amino acids are displayed for different variants. VP1 comprises residues 1–297 whereas VP2 consists of residues 298–542. BC, EF and GH loops of VP1 are labelled accordingly.
(TIF)

S1 Table Non-synonymous mutations related to heparin binding detected from different organ samples of IEE-infected mice. (DOCX)

S2 Table Comparison of EV-A71 isolate sequences of primary specimens and passaged isolates. Strong heparin binders (denoted with asterisks) were more frequently identified from sequencing of passaged EV-A71 (at least one passage) than from direct sequencing of primary specimens, suggesting that the virus isolates have undergone heparin-binding adaptation in cell culture (*P* = 0.00012, chi-square test).
(DOCX)

S3 Table Primer sequences used for RT-PCR and qRT-PCR. Primer sets used for EV-A71 VP1 sequencing and qRT-PCR are shown.
(DOCX)

S1 Appendix Details of published sequences obtained from direct sequencing of clinical specimens and sequencing from cell cultures. (PDF)

S1 Text Establishment of *in vitro* blood-brain barrier model. (DOCX)
