# Supplementary figures and images for: Electrostatic interactions at the five-fold axis alter heparin-binding phenotype and drive enterovirus A71 virulence in mice
Source: PLoS Pathog. 2019 Nov 15;15(11):e1007863. doi: 10.1371/journal.ppat.1007863 (PMC6881073; doi:10.1371/journal.ppat.1007863)

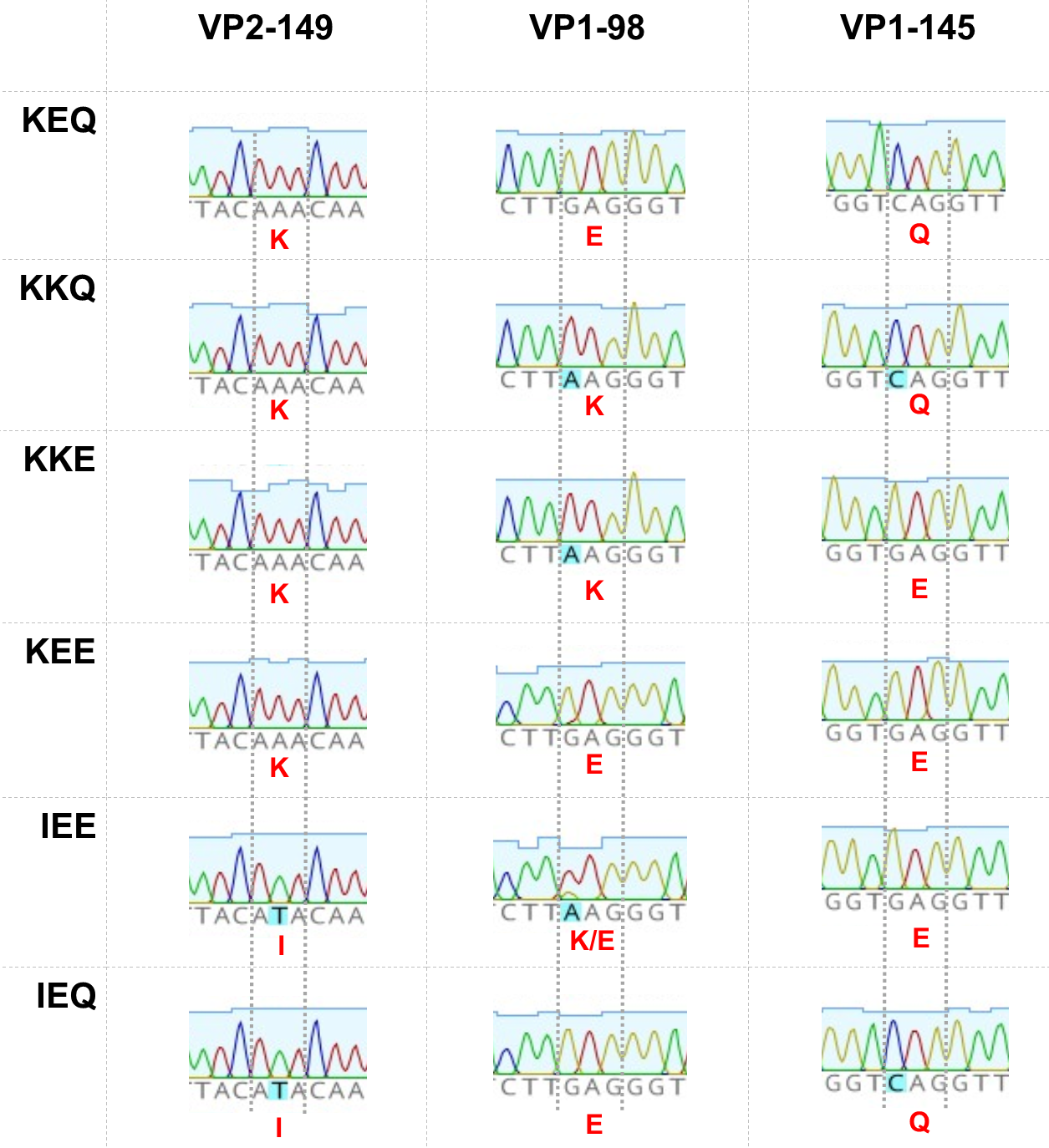

Supplement: S1 Fig — (TIF) [file ppat.1007863.s001.tif]

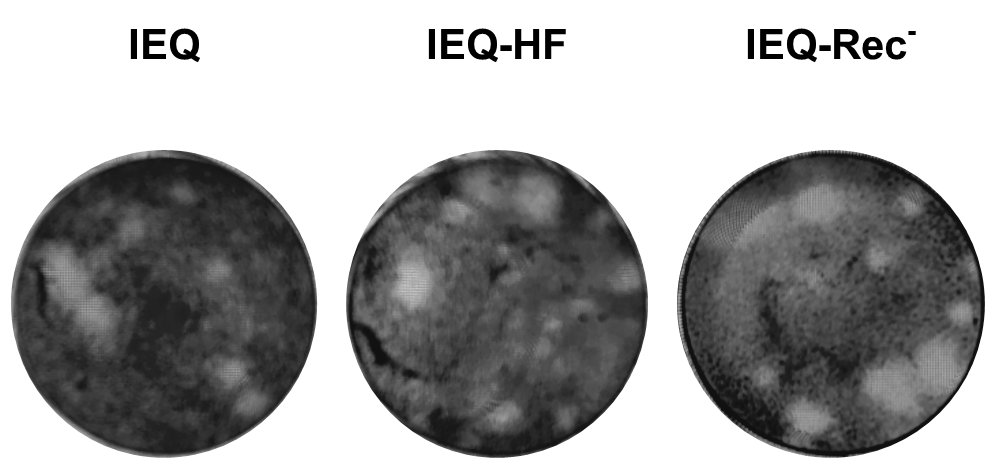

Supplement: S2 Fig — (TIF) [file ppat.1007863.s002.tif]

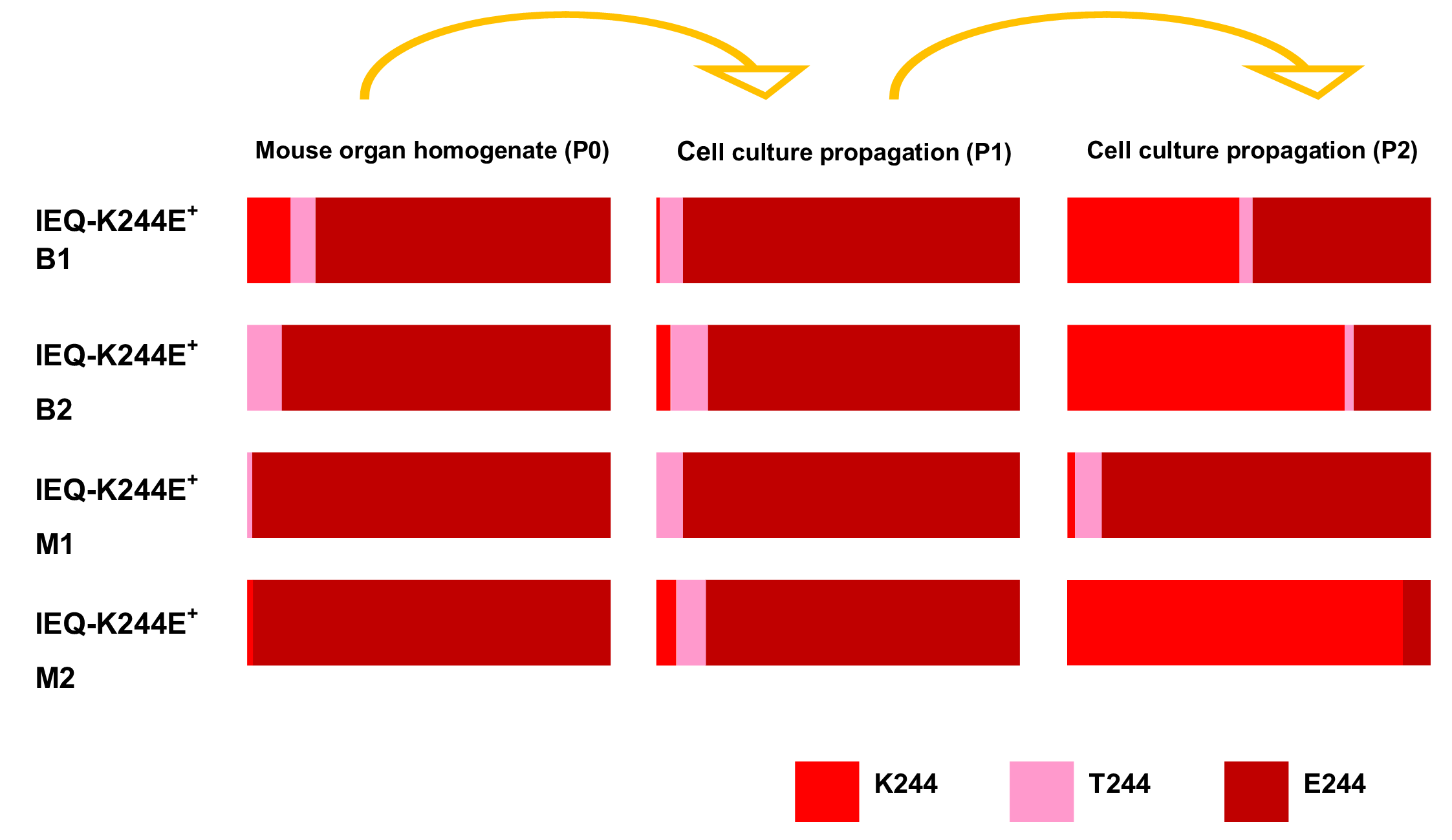

Supplement: S3 Fig — A sequential reversion of VP1-244E to 244K was observed in IEQ-244E+ after cell culture propagation. Note that the 244T variant was also present in the mixed population. The mixed populations of K244 (red), T244 (pink) and E244 (maroon) are shown in different proportions. Only variants with frequency >1% are reported. Note that samples B1 at P0 and B2 at P0 had poor sequencing coverages (between 110–5100). M indicates muscle, B indicates brain. (TIF) [file ppat.1007863.s003.tif]

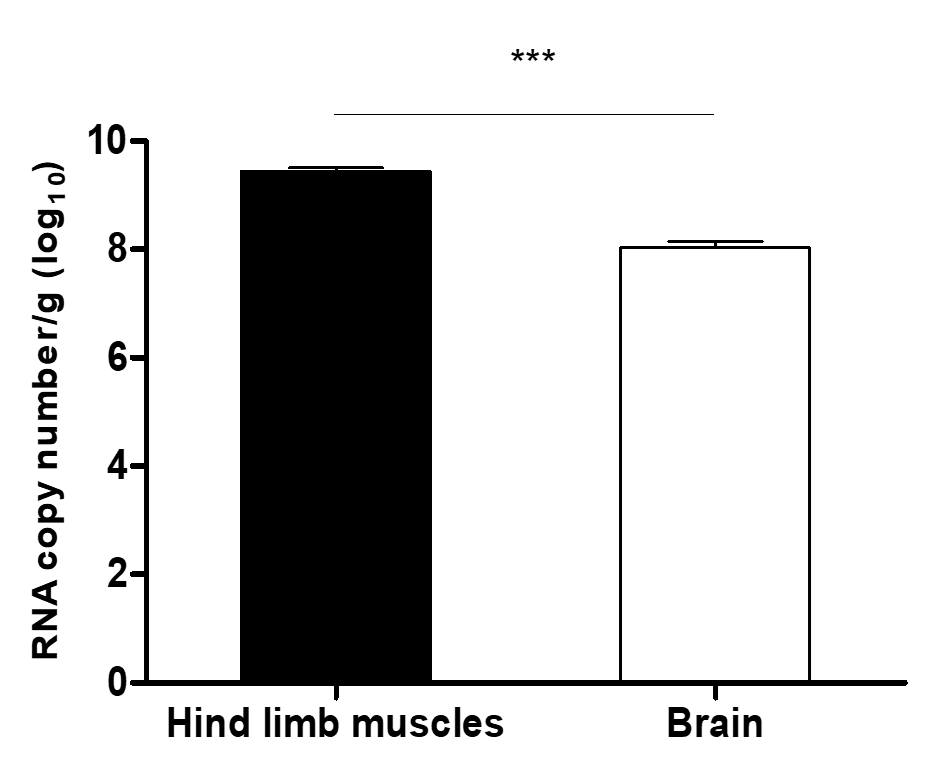

Supplement: S4 Fig — One-day old suckling mice (n = 3) were infected with IEE through i.c. route of administration. At day 4 post-infection, muscles and brains were harvested and viral loads were quantitated using qRT-PCR. Significant comparisons are labelled *** (P < 0.001). (TIF) [file ppat.1007863.s004.tif]

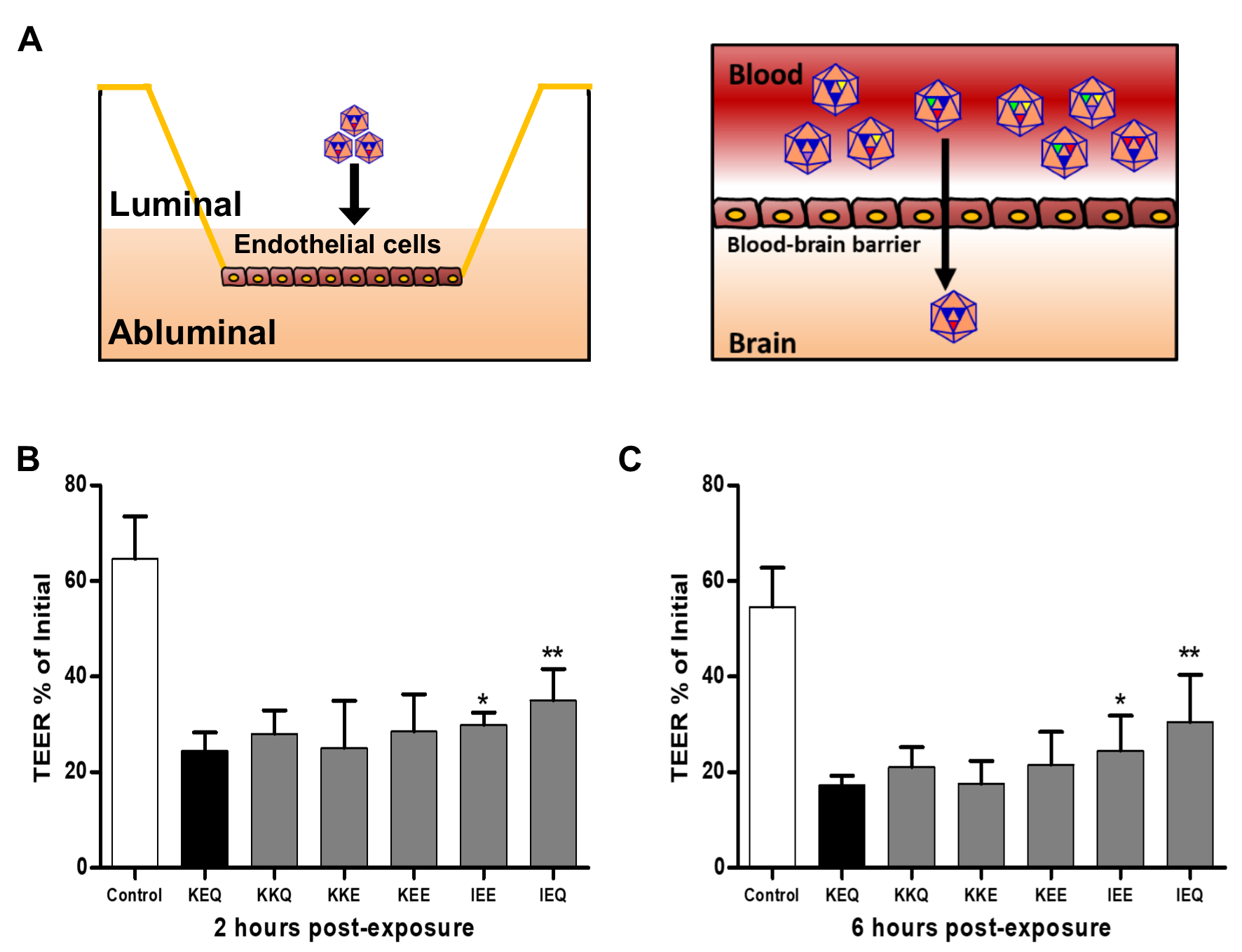

Supplement: S5 Fig — Illustration of the porcine in vitro BBB model which simulates the movement of virus particles through an in vivo BBB, in which the luminal side represents the blood capillary while the abluminal compartment represents the brain (A). The in vitro model was exposed to different EV-A71 variants with titer of 1 × 10^5^ PFU. The BBB permeability induced by EV-A71 variants were assessed in terms of transendothelial electrical resistance (TEER), with a greater reduction of TEER indicating greater permeability of BBB through tight junction leakages. The TEER was recorded at 2 hours (B) and 6 hours post-exposure (C) along with non-infected cell controls (white bars) and normalized with TEER values measured before virus exposure. Results are presented as mean ± SD (n = 6). Significant differences between viral variants and WT (black bars) are labelled as * (P < 0.05) and **(P < 0.01), using the Student’s t test. (TIF) [file ppat.1007863.s005.tif]

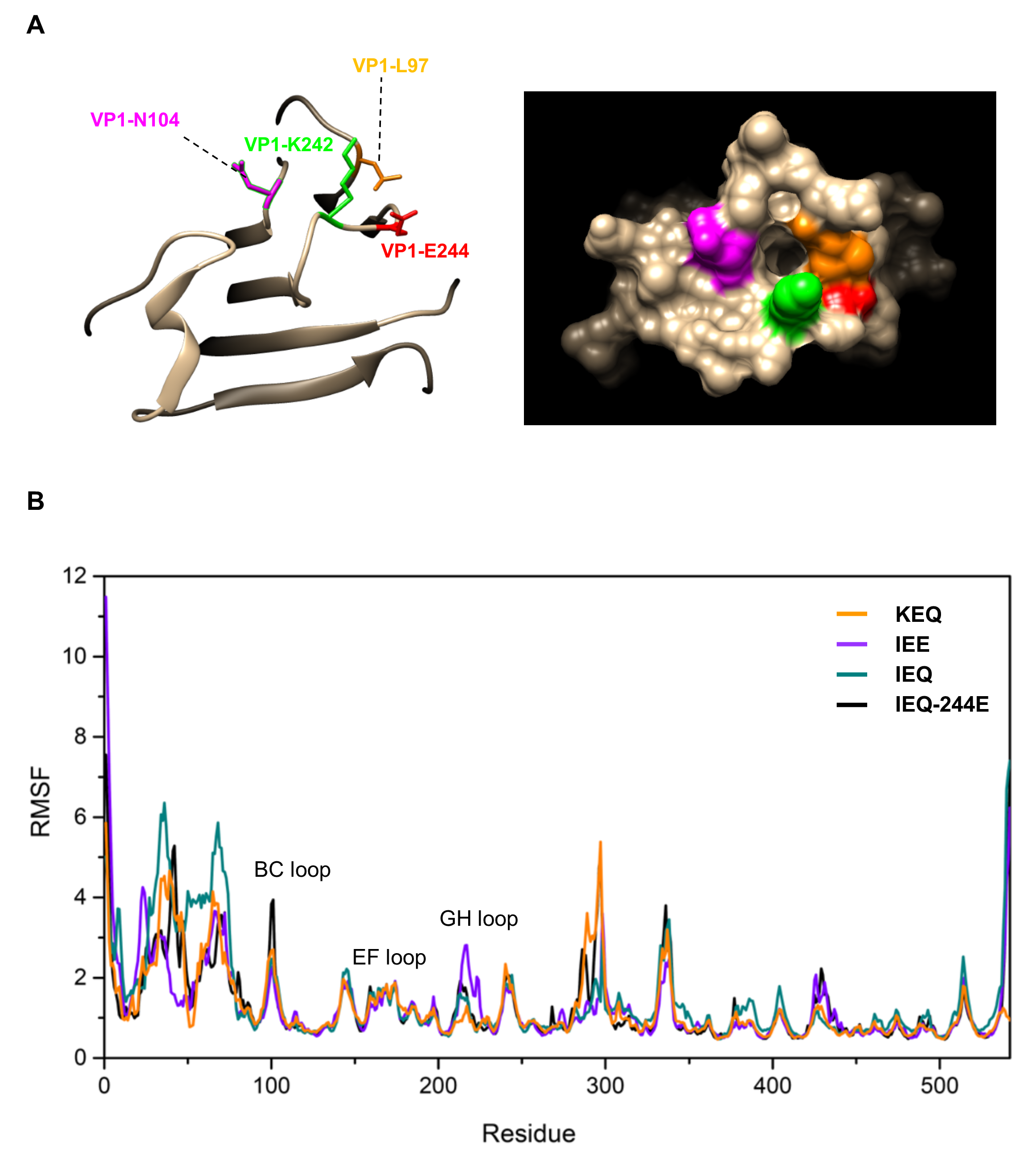

Supplement: S6 Fig — (A) Structural modelling of VP1 amino acid residues of IEQ-244E (left panel). Each important amino acid is labelled with different colors: VP1-244E in red, VP1-244K in green, VP1-97L in orange and VP1-104N in magenta. Note that VP1-145E is not visible from this angle. The surface of IEQ-244E (right panel) is displayed corresponding to the structural model. (B) Root mean square fluctuation (RMSF) value of VP1 and VP2 amino acids are displayed for different variants. VP1 comprises residues 1–297 whereas VP2 consists of residues 298–542. BC, EF and GH loops of VP1 are labelled accordingly. (TIF) [file ppat.1007863.s006.tif]
