# Supplementary material for: Electrostatic interactions at the five-fold axis alter heparin-binding phenotype and drive enterovirus A71 virulence in mice
Source: PLoS Pathog. 2019 Nov 15;15(11):e1007863. doi: 10.1371/journal.ppat.1007863 (PMC6881073; doi:10.1371/journal.ppat.1007863)
Supplement: S1 Table — (DOCX) [file ppat.1007863.s007.docx]

**S1 Table: Non-synonymous mutations related to heparin binding detected from different organ samples of IEE-infected mice**

|  | **97 L** | | **98 E** | | **104 N** | | **145 E** | | **167 E** | | **244 K** | |  |
| --- | --- | --- | --- | --- | --- | --- | --- | --- | --- | --- | --- | --- | --- |
|  | Mutation | Frequency | Mutation | Frequency | Mutation | Frequency | Mutation | Frequency | Mutation | Frequency | Mutation | Frequency | |
| **Input** | - | - | E98K | 65.6 | - | - | E145Q | 1.2 | - | - | - | - |  |
| **B2** | - | - | E98K | 1.0 | - | - | - | - | - | - | - | - |  |
| **M2** | - | - | - | - | - | - | E145Q | 4.97 | - | - | K244E | 6.22 |  |
| **B4** | - | - | - | - | - | - | - | - | - | - | - | - |  |
| **M4** | - | - | - | - | N104S | 1.1 | - | - | - | - | - | - |  |
| **B6** | - | - | - | - | - | - | - | - | - | - | - | - |  |
| **M6** | - | - | - | - | - | - | - | - | - | - | - | - |  |

Note: - indicates no variation >1% was observed. M indicates muscle, B indicates brain. Note that samples M2 and M4 had sequencing coverages between 3000-6000 at the respective mutations.
