# Supplementary material for: Electrostatic interactions at the five-fold axis alter heparin-binding phenotype and drive enterovirus A71 virulence in mice
Source: PLoS Pathog. 2019 Nov 15;15(11):e1007863. doi: 10.1371/journal.ppat.1007863 (PMC6881073; doi:10.1371/journal.ppat.1007863)
Supplement: S2 Table — Strong heparin binders (denoted with asterisks) were more frequently identified from sequencing of passaged EV-A71 (at least one passage) than from direct sequencing of primary specimens, suggesting that the virus isolates have undergone heparin-binding adaptation in cell culture (P = 0.00012, chi-square test). (DOCX) [file ppat.1007863.s008.docx]

**S2 Table. Comparison of EV-A71 isolate sequences of primary specimens and passaged isolates**

| **Sequence  combination** | **Sequencing approach** | | | |
| --- | --- | --- | --- | --- |
|  | Direct sequencing | | Sequencing from tissue  culture propagation | |
|  | Number of  sequences | Percentage (%) | Number of  sequences | Percentage (%) |
| EE | 187 | 89.05 | 142 | 69.95 |
| EQ* | 8 | 3.81 | 9 | 4.43 |
| EG* | 2 | 0.95 | 17 | 8.37 |
| KE* | 12 | 5.71 | 32 | 15.76 |
| Others | 1 | 0.48 | 3 | 1.48 |
| Total | 210 | 100.00 | 203 | 100.00 |
| Chi-square test | χ^2^ = 28.0363 *P* = 0.000012 | | | |

Note: Strong heparin binders (denoted with *) were more frequently identified from sequencing of passaged EV-A71 than from direct sequencing of primary specimens suggesting that the virus isolates have undergone heparin-binding adaptation in cell culture (*P* = 0.000012, chi-square test).
