# Supplementary material for: Electrostatic interactions at the five-fold axis alter heparin-binding phenotype and drive enterovirus A71 virulence in mice
Source: PLoS Pathog. 2019 Nov 15;15(11):e1007863. doi: 10.1371/journal.ppat.1007863 (PMC6881073; doi:10.1371/journal.ppat.1007863)
Supplement: S3 Table — Primer sets used for EV-A71 VP1 sequencing and qRT-PCR are shown. (DOCX) [file ppat.1007863.s009.docx]

**S3 Table. Primer sequences used for VP1 RT-PCR and qRT-PCR.**

| **Primer** | **Sequence (5’ to 3’)** |
| --- | --- |
| EV71-VP1 F | GCACTAGCGGCAGCCCAGAAGAA |
| EV71-VP1 R | GAGCTATCTTCCCAGACGAGGTTC |
| EV71-real time F | GAGCTCTATAGGAGATAGTGTGAGTAGGG |
| EV71-real time R | ATGACTGCTCACCTGCGTGTT |
| EV71-real time probe | 6-carboxyfluorescein (FAM)-ACTTACCCA/ZEN/GGCCCTGCCAGCTCG-Iowa Black FQ |
