# Supplementary material for: Electrostatic interactions at the five-fold axis alter heparin-binding phenotype and drive enterovirus A71 virulence in mice
Source: PLoS Pathog. 2019 Nov 15;15(11):e1007863. doi: 10.1371/journal.ppat.1007863 (PMC6881073; doi:10.1371/journal.ppat.1007863)
Supplement: S1 Appendix — (PDF) [file ppat.1007863.s010.pdf]

Direct sequencing

NCBI	Sample type	Clinical outcome*	Sequence (VP1-98 + VP1-145)	Reference
KP691643	Throat swab	Mild	EE	[1]
KP691644	Throat swab	Mild	EE	
KP691645	Throat swab	Mild	EE	
KP691646	Throat swab	Mild	EE	
KP691647	Throat swab	Mild	EE	
KP691648	Throat swab	Mild	EE	
KP691649	Throat swab	Mild	EE	
KP691650	Throat swab	Mild	EE	
KP691651	Throat swab	Mild	EE	
KP691652	Throat swab	Mild	EE	
KP691653	Throat swab	Mild	EE	
KP691654	Throat swab	Mild	EE	
KP691655	Throat swab	Mild	EE	
KP691656	Throat swab	Mild	EE	
KP691657	Throat swab	Mild	EE	
KP691658	Throat swab	Mild	EE	
KP691659	Throat swab	Mild	EE	
KP691660	Throat swab	Mild	EE	
KP691661	Throat swab	Mild	EE	
KP691662	Throat swab	Mild	EE	
KP691663	Throat swab	Mild	EE	
KP691664	Throat swab	Mild	EE	
KP691665	Throat swab	Mild	EE	
KP691666	Throat swab	Mild	EE	
JQ419491	Throat swab	Mild	EE	[2]
JQ419492	Throat swab	Mild	EE	
JQ419493	Throat swab	Mild	KE	
JQ419494	Throat swab	Mild	EE	
JQ419495	Throat swab	Mild	EE	
JQ419496	Throat swab	Mild	EE	
JQ419497	Throat swab	Mild	KE	
JQ419498	Throat swab	Mild	KE	
JQ419499	Throat swab	Severe	EE	
JQ766195	Throat swab, fecal swab	Severe	EE	[3]
KY796193	Throat swab	Mild	EE	[4]
KT717067	Stool sample	Mild	EE	[5]
KT717068	Stool sample	Mild	EE	
KJ156339	Stool sample	N.D.	EE	[6]

KJ156340	Stool sample		EE	
KJ156341	Stool sample		EE	
KJ156342	Stool sample		EE	
KJ156343	Throat swab		EE	
KJ784497	Throat swab		EE	
KJ784498	Throat swab		EE	
KJ784499	Throat swab		EE	
KJ784500	Throat swab		EE	
KY041858	Nasal swab	N.D.	EE	[7]
KY041859	Nasal swab		EE	
KY041860	Nasal swab		EE	
KY041861	Nasal swab		EE	
KY041862	Nasal swab		EE	
KY041863	Nasal swab		EE	
KY041864	Nasal swab		EE	
KP171496	Throat swab	Mild or severe	EE	[8]
KP171497	Throat swab		EE	
KP171498	Throat swab		EE	
KP171499	Throat swab		EE	
KP171500	Throat swab		EE	
KP171501	Throat swab		EE	
KP171502	Throat swab		EE	
KP171503	Throat swab		EE	
KP171504	Throat swab		EE	
KP171505	Throat swab		EE	
KP171506	Throat swab		EE	
KP171507	Throat swab		EE	
KP171508	Throat swab		EE	
KP171509	Throat swab		EE	
HQ828086	Intestinal content	Fatal	EE	[9]
GQ231934	Throat swab, rectal swab	Mild	EE	[10]
GQ231935	Throat swab, rectal swab	Mild	EE	
GQ231936	Throat swab, rectal swab	Mild	EE	
GQ231937	Throat swab, rectal swab	Severe	EE	
GQ231939	Throat swab, rectal swab	Severe	EE	
GQ231940	Throat swab, rectal swab	Mild	EE	
GQ231941	Throat swab, rectal swab	Severe	EQ	

GQ231942	Throat swab, rectal swab	Severe	EE	
GQ231943	Throat swab, rectal swab	Severe	EE	
EU414333	CSF	Severe	EE	[11]
JX990970	N.D.	N.D.	EE	[12]
JX990971	N.D.		EE	
JX990972	N.D.		KE	
JX990973	N.D.		EE	
JX990974	N.D.		EE	
JX990975	N.D.		EE	
JX990976	N.D.		KE	
JX990977	N.D.		KE	
JX990978	N.D.		KE	
JX990979	N.D.		KE	
JX990980	N.D.		KE	
JX990981	N.D.		EE	
JX990982	N.D.		KE	
JX990983	N.D.		EE	
JX990984	N.D.		EE	
JX990985	N.D.		KE	
JX990986	N.D.		EE	
JX990987	N.D.		EE	
JX990988	N.D.		EE	
JX990989	N.D.		KE	
JX990990	N.D.		EE	
JX990991	N.D.		EE	
JX990992	N.D.		EQ	
JX990993	N.D.		EE	
JX990994	N.D.		EG	
JX990995	N.D.		EE	
JX990996	N.D.		EE	
JX990997	N.D.		EE	
JX990998	N.D.		EE	
JX990999	N.D.		EE	
JX991000	N.D.		EQ	
JX991001	N.D.		EE	
JX991002	N.D.		EQ	
JX991003	N.D.		EQ	
JX991004	N.D.		EQ	
JX991005	N.D.		EQ	
JX991006	N.D.		EE	
JX991007	N.D.		EE	

JX991008	N.D.		EE	
JX991009	N.D.		EE	
JX991010	N.D.		EE	
JX991011	N.D.		EG	
JX991012	N.D.		EE	
JX991013	N.D.		EE	
JX991014	N.D.		EE	
JX991015	N.D.		EE	
JX991016	N.D.		EE	
JX991017	N.D.		EE	
JX991018	N.D.		EE	
JX991019	N.D.		EE	
JX991020	N.D.		EE	
JX991021	N.D.		EE	
JX991022	N.D.		EE	
JX991023	N.D.		EE	
JX991024	N.D.		EE	
JX991025	N.D.		EE	
JX991026	N.D.		EE	
JX991027	N.D.		EE	
JX991028	N.D.		EE	
JX991029	N.D.		EE	
JX991030	N.D.		EE	
JX991031	N.D.		EE	
JX991032	N.D.		EE	
JX991033	N.D.		EE	
JX991034	N.D.		EE	
JX991035	N.D.		EE	
JX991036	N.D.		EE	
JX991037	N.D.		EE	
JX991038	N.D.		EE	
JX991039	N.D.		EE	
JX991040	N.D.		EE	
JX991041	N.D.		EE	
JX991042	N.D.		EE	
JN944847	Throat swab	Mild or severe	EE	[13]
JN944848	Throat swab		EE	
JN944849	Throat swab		EE	
JN944850	Throat swab		EE	
JN944851	Throat swab		EE	
JN944852	Throat swab		EE	
JN944853	Throat swab		EE	
JN944854	Throat swab		EE	

JN944855	Throat swab		EE	
JN944856	Throat swab		EE	
JN944857	Throat swab		EE	
JN944858	Throat swab		EE	
JN944859	Throat swab		EE	
JN944860	Throat swab		EE	
JN944861	Throat swab		EE	
JN944862	Throat swab		EE	
JN944863	Throat swab		EE	
JN944864	Throat swab		EE	
JN944865	Throat swab		EE	
JN944866	Throat swab		EE	
JN944867	Throat swab		EE	
JN944868	Throat swab		EE	
JN944869	Throat swab		EE	
JN944870	Throat swab		EE	
JN944871	Throat swab		EE	
JN944872	Throat swab		EE	
JN944873	Throat swab		EE	
JN944874	Throat swab		EE	
JN944875	Throat swab		EE	
JN944876	Throat swab		EE	
JN944877	Throat swab		EE	
JN944878	Throat swab		EE	
JN944879	Throat swab		EE	
JN944880	Throat swab		EE	
JN944881	Throat swab		EE	
JN944882	Throat swab		EE	
JN944883	Throat swab		EE	
JN944884	Throat swab		EE	
JN944885	Throat swab		EE	
JN944886	Throat swab		EE	
JN944887	Throat swab		EE	
JN944888	Throat swab		EE	
JN944889	Throat swab		EE	
JN944890	Throat swab		EE	
JN944891	Throat swab		EE	
JN944892	Throat swab		EE	
JN944893	Throat swab		EE	
JN944894	Throat swab		EE	
JN944895	Throat swab		EE	

Sequencing from cell culture adaptation

NCBI	Sample type	Cell	Clinical outcome*	Sequence (VP1-98 + VP1-145)	Reference
MG773122	Throat swab	RD	Mild	EE	[14]
MG773123	Throat swab	RD	Mild	EE	
MG773124	Throat swab	RD	Mild	EE	
MG773125	Throat swab	RD	Mild	EE	
MG773126	Throat swab	RD	Mild	EE	
KX197414	Throat swab, rectal swab	Vero	Severe	EE	[15]
KX197415	Throat swab, rectal swab	Vero	Severe	EE	
KX197416	Throat swab, rectal swab	Vero	Severe	EE	
KX197417	Throat swab, rectal swab	Vero	Severe	EE	
KX197418	Throat swab, rectal swab	Vero	Mild	EE	
KX197419	Throat swab, rectal swab	Vero	Mild	EE	
KX197420	Serum, throat and rectal swabs	Vero	Mild	EE	
KX197421	Serum and rectal swab / BAL, throat and rectal swabs	Vero	Severe	EE	
KX197422	Throat and rectal swabs	Vero	Severe	EE	
KX197455	Throat and rectal swabs	Vero	Severe	EE	
KX197456	Throat and rectal swabs	Vero	Mild	EE	
KX197457	Throat and rectal swabs	Vero	Mild	EE	
KX197458	Serum, throat and rectal swabs	Vero	Severe	EE	
KX197459	BAL, throat and rectal swabs	Vero	Mild	EE	
KX197460	Throat and rectal swabs	Vero	Mild	EE	
KX197461	CSF, throat and rectal swabs	Vero	Severe	EE	

KX197462	Throat and rectal swabs	Vero	Severe	EE	
KX197463	CSF, throat and rectal swabs / Throat swab	Vero	Severe	EE	
KX197464	Serum, throat and rectal swabs	Vero	Severe	EE	
KX197465	Serum, throat and rectal swabs	Vero	Severe	EE	
EU868611	Throat swabs, nasal swabs, rectal swabs, saliva, urine, and blood	RD	Mild	EE	[16]
FJ461781	Throat swabs, nasal swabs, rectal swabs, saliva, urine, and blood	RD	Mild	EE	
FJ461782	Throat swabs, nasal swabs, rectal swabs, saliva, urine, and blood	RD	Mild	EE	
FJ461783	Throat swabs, nasal swabs, rectal swabs, saliva, urine, and blood	RD	Mild	EE	
FJ461784	Throat swabs, nasal swabs, rectal swabs, saliva, urine, and blood	RD	Mild	EE	
FJ461785	Throat swabs, nasal swabs, rectal swabs, saliva, urine, and blood	RD	Mild	EE	
FJ461786	Throat swabs, nasal swabs, rectal swabs, saliva, urine, and blood	RD	Mild	EE	
FJ461787	Throat swabs, nasal swabs, rectal swabs, saliva, urine, and blood	RD	Mild	EE	

FJ461788	Throat swabs, nasal swabs, rectal swabs, saliva, urine, and blood	RD	Mild	EE	
FJ461789	Throat swabs, nasal swabs, rectal swabs, saliva, urine, and blood	RD	Mild	EE	
JN835312	Pharyngeal swab	BGM	Mild	EE	[17]
GQ117124	Throat swab, vesicular fluid	RD	Mild	KE	[18]
GQ117125	Throat swab, vesicular fluid	RD	Mild	KE	
GQ117126	Throat swab, vesicular fluid	RD	Mild	KE	
GQ117127	Throat swab, vesicular fluid	RD	Mild	KE	
GQ117128	Throat swab, vesicular fluid	RD	Mild	KE	
KU645333	CSF, throat swab, fecal specimen	RD, MRC-5, FrHK	Severe	EE	[19]
KU645334	CSF, throat swab, fecal specimen	RD, MRC-5, FrHK	Severe	EE	
KU645335	CSF, throat swab, fecal specimen	RD, MRC-5, FrHK	Severe	EE	
KU645336	CSF, throat swab, fecal specimen	RD, MRC-5, FrHK	Severe	EE	
KU645337	CSF, throat swab, fecal specimen	RD, MRC-5, FrHK	Severe	EE	
KU645338	CSF, throat swab, fecal specimen	RD, MRC-5, FrHK	Severe	EE	
AB213618	Throat swab, nasal swab	HEF, GMK	Mild	EE	[20]
AB550333	Throat swab	RD	Mild	EG	[21]
AB550335	Rectal swab	Vero	Fatal	EG	
AB550337	Stool	Vero	Fatal	EG	
AB550339	Nasopharyngeal swab	RD	Mild	EQ	
AB550341	Stool	RD	Mild	EQ	
AY207611	Vesicle swab	Vero, RD	Mild	EE	[22]

AY207612	Vesicle swab	Vero, RD	Mild	EE	
AY207613	Vesicle swab	Vero, RD	Mild	EE	
AY207614	Oral swab	Vero, RD	Mild	EE	
AY207615	Vesicle swab	Vero, RD	Mild	EE	
AY207616	Stool	Vero, RD	Mild	EE	
AY207617	Throat swab	Vero, RD	Mild	EE	
AY207618	Rectal swab	Vero, RD	Mild	EE	
AY207619	Vesicle swab	Vero, RD	Mild	EE	
AY207620	Throat swab	Vero, RD	Mild	EE	
AY207621	Throat swab	Vero, RD	Mild	EE	
AY207622	Rectal swab	Vero, RD	Mild	EE	
AY207623	Rectal swab	Vero, RD	Mild	EE	
AY207624	Throat swab	Vero, RD	Mild	EE	
AY207625	Throat swab	Vero, RD	Mild	EE	
AY207626	Rectal swab	Vero, RD	Mild	EE	
AY207627	Vesicle swab	Vero, RD	Mild	EE	
AY207628	Throat swab	Vero, RD	Mild	KE	
AY207629	Throat swab	Vero, RD	Mild	KE	
AY207630	Throat swab	Vero, RD	Severe	KE	
AY207631	Rectal swab	Vero, RD	Mild	KE	
AY207632	Throat swab	Vero, RD	Mild	EE	
AY207633	Vesicle swab	Vero, RD	Mild	EE	
AY207634	Throat swab	Vero, RD	Mild	EE	
AY207635	Ulcer swab	Vero, RD	Mild	EE	
AY207636	Throat swab	Vero, RD	Mild	EG	
AY207637	Throat swab	Vero, RD	Mild	KE	
AY207638	Throat swab	Vero, RD	Mild	KE	
AY207639	Throat swab	Vero, RD	Severe	KE	
AY207640	Rectal swab	Vero, RD	Mild	KE	
AY207641	Oral swab	Vero, RD	Mild	EG	
AY207642	Cerebellum	Vero, RD	Severe	EG	
AY207643	Spinal cord	Vero, RD	Severe	EQ	
AY207644	Stool	Vero, RD	Mild	EG	
AY207645	Stool	Vero, RD	Mild	EG	
AY207646	Brainstem	Vero, RD	Severe	KE	
AY207647	Stool	Vero, RD	Mild	EE	
AY207648	Spinal cord	Vero, RD	Severe	EG	
AY207649	Left brain	Vero, RD	Myocarditis	KE	
AY207650	Rectal swab	Vero, RD	Mild	EE	
AY207651	Stool	Vero, RD	Mild	EE	
AY207652	Rectal swab	Vero, RD	Severe	KE	
AY207653	Vesicle swab	Vero, RD	Severe	KE	
HQ129932	Vesicular fluid	RD		EE	[23]

FJ606447	Vesicular fluid	RD	Mild and severe	EE	
FJ606448	Vesicular fluid	RD		EE	
FJ606449	Vesicular fluid	RD		EE	
FJ606450	Vesicular fluid	RD		EE	
JN544418	Throat swab	Vero	Fatal	EQ	[24]
AM396587	CSF, rectal swab, stool	Vero	Fatal	KE	[25]
AM396584	CSF, rectal swab, stool	Vero	Mild	EE	
AM396588	CSF, rectal swab, stool	Vero	Mild	EE	
AM396586	CSF, rectal swab and stool	Vero	Mild	KE	
EU812515	Throat swab	Vero	Mild	EE	[26]
FJ439769	Pharyngeal swab	RD	Mild	EE	[27]
FJ828519	Stool	N.D.	Mild	EE	[28]
JN256059	Vesicular swab, throat swab and stool	RD	Mild	KE	[29]
JN256060	Vesicular swab, throat swab and stool	RD	Mild	EE	
JN256061	Vesicular swab, throat swab and stool	RD	Mild	EE	
JN256062	Vesicular swab, throat swab and stool	RD	Mild	EE	
JN256063	Vesicular swab, throat swab and stool	RD	Mild	EE	
JN256064	Vesicular swab, throat swab and stool	RD	Mild	EE	
JN256065	Vesicular swab, throat swab and stool	RD	Mild	EE	
JN256066	Vesicular swab, throat swab and stool	RD	Mild	EE	
JN256067	Vesicular swab, throat swab and stool	RD	Mild	EE	
JN256068	Vesicular swab, throat swab and stool	RD	Mild	EE	
HM037792	Feces	RD	Fatal	EE	[30]
HM037793	Feces	RD	Mild	EE	

HM037794	Feces	RD	Severe	EE	
HM037795	Rectal swab	RD	Severe	EE	
HM037796	Feces	RD	Severe	EE	
HM037797	Feces	RD	Mild	EE	
HM037798	Vesicle fluid	RD	Mild	EE	
HM037799	Rectal swab	RD	Mild	EE	
HM037800	Feces	RD	Fatal	EG	
HM037801	Throat swab	RD	Severe	EE	
HM037802	Feces	RD	Mild	KE	
HM037803	Rectal swab	RD	Severe	EQ	
HM037804	Feces	RD	Severe	EE	
HM037805	Throat swab	RD	Severe	EE	
HM037806	Feces	RD	Mild	EK	
HM037807	Feces	RD	Fatal	EE	
HM037808	Throat swab	RD	Fatal	EE	
GU196833	Anal swab	Vero, RD	Mild	KE	[31]
GQ994992	Anal swab	Vero, RD	Mild	KE	
GQ994989	Stool	Vero, RD	Mild	KE	
GQ994990	Stool	Vero, RD	Mild	KE	
GQ994991	Anal swab	Vero, RD	Mild	KE	
GQ994988	Throat swab	Vero, RD	Mild	KE	
DQ341354	Vesicle, throat swab, rectal swab, stool and CSF	N.D.	Mild or severe	EQ	[32]
DQ341358	Vesicle, throat swab, rectal swab, stool and CSF	N.D.		EE	
DQ341359	Vesicle, throat swab, rectal swab, stool and CSF	N.D.		EE	
DQ341361	Vesicle, throat swab, rectal swab, stool and CSF	N.D.		EE	
DQ341364	Vesicle, throat swab, rectal swab, stool and CSF	N.D.		EE	
DQ452074	Vesicle, throat swab, rectal swab, stool and CSF	N.D.		EE	
AF376065	CSF	N.D.	Severe	EE	

AF376105	Stool	N.D.	Severe	EG	
AF376106	CSF	N.D.	Severe	KE	
AF376109	CSF	N.D.	Severe	KE	
AF376110	CSF	N.D.	Severe	KE	
AF376111	N.D.	N.D.	N.D.	EQ	
AF376116	Stool	N.D.	Severe	EG	
AF376125	Throat swab	N.D.	Severe	EG	
JX244182	Stool	RD	Mild	EG	[33]
JX244183	Stool	RD	Mild	EG	
JX244184	Stool	RD	Mild	EE	
JX244185	Stool	RD	Severe	EE	
JX244186	Anal swab	RD	Fatal	KE	
JX244187	Anal swab	RD	Severe	KQ	
KC436265	Stool	RD	Mild	EE	[34]
KC436266	Stool	RD	Mild	EE	
KC436267	Stool	RD	Mild	EE	
KC436268	Stool	RD	Mild	EE	
KC436269	Rectal swab	RD	Mild	EE	
KC436270	Stool	RD	Mild	EE	
KC436271	Stool	RD	Mild	EE	
KC436272	Stool	RD	Mild	EE	
KC436273	Stool	RD	Mild	EE	
KC436274	Throat swab	RD	Mild	EE	
KC436275	Stool	RD	Mild	EE	
KC436276	Stool	RD	Mild	EE	
KC436277	Stool	RD	Mild	EE	
KC436278	Stool	RD	Mild	EE	
KC436279	Throat swab	RD	Mild	EE	
KC436280	Stool	RD	Mild	EE	
KC436281	Stool	RD	Mild	EE	
KC436282	Stool	RD	Mild	EE	
KC436283	Stool	RD	Mild	EE	
KC436284	Nasopharyngeal aspirate	RD	Mild	EE	
KC436285	Stool	RD	Mild	EE	
KC436286	Nasopharyngeal aspirate	RD	Mild	EE	
AF009523	N.D.	RD	Severe	EE	[35]
AF009525	N.D.	RD	Severe	EE	
AF009526	N.D.	RD	Severe	EQ	
AF009527	N.D.	RD	Severe	EG	
AF009539	N.D.	RD	Severe	EE	
AF009542	N.D.	RD	Severe	EE	

AF009546	N.D.	RD	Severe	EE	
AF009549	N.D.	RD	Severe	EE	
AF135883	N.D.	RD	Severe	EE	
AF135884	N.D.	RD	Severe	EE	
AF135885	N.D.	RD	Severe	EE	
AF135886	N.D.	RD	Severe	EE	
AF135893	N.D.	RD	Severe	EG	
AF135895	N.D.	RD	Severe	KE	
AF135899	N.D.	RD	Severe	EE	
AF135938	N.D.	RD	Severe	EQ	
AF135940	N.D.	RD	Severe	EA	
AF135948	N.D.	RD	Severe	EE	
AF135950	N.D.	RD	Severe	EE	

*N.D. indicates not determined.

References

1. Tan le V, Tuyen NT, Thanh TT, Ngan TT, Van HM, Sabanathan S, et al. A generic assay for whole-genome amplification and deep sequencing of enterovirus A71. J Virol Methods. 2015;215-216:30-6. Epub 2015/02/24. doi: 10.1016/j.jviromet.2015.02.011. PubMed PMID: 25704598; PubMed Central PMCID: PMCPMC4374682.
2. Liu Y, Fu C, Wu S, Chen X, Shi Y, Zhou B, et al. A novel finding for enterovirus virulence from the capsid protein VP1 of EV71 circulating in mainland China. Virus Genes. 2014;48(2):260-72. Epub 2014/01/21. doi: 10.1007/s11262-014-1035-2. PubMed PMID: 24442718.
3. Li J, Huo X, Dai Y, Yang Z, Lei Y, Jiang Y, et al. Evidences for intertypic and intratypic recombinant events in EV71 of hand, foot and mouth disease during an epidemic in Hubei Province, China, 2011. Virus Res. 2012;169(1):195-202. Epub 2012/08/28. doi: 10.1016/j.virusres.2012.07.028. PubMed PMID: 22922556.
4. Ganorkar NN, Patil PR, Tikute SS, Gopalkrishna V. Genetic characterization of enterovirus strains identified in Hand, Foot and Mouth Disease (HFMD): Emergence of B1c, C1 subgenotypes, E2 sublineage of CVA16, EV71 and CVA6 strains in India. Infect Genet Evol. 2017;54:192-9. Epub 2017/06/05. doi: 10.1016/j.meegid.2017.05.024. PubMed PMID: 28577914.
5. Faleye TO, Adewumi MO, Coker BA, Nudamajo FY, Adeniji JA. Direct detection and identification of enteroviruses from faeces of healthy Nigerian children using a cell-culture independent RT-seminested PCR assay. Adv Virol. 2016;2016:1412838. Epub 2016/04/19. doi: 10.1155/2016/1412838. PubMed PMID: 27087810; PubMed Central PMCID: PMCPMC4818813.
6. Chen JF, Zhang RS, Ou XH, Chen FM, Sun BC. The role of enterovirus 71 and coxsackievirus A strains in a large outbreak of hand, foot, and mouth disease in 2012 in Changsha, China. Int J Infect Dis. 2014;28:17-25. Epub 2014/09/23. doi: 10.1016/j.ijid.2014.07.024. PubMed PMID: 25236389.
7. Wiyatno A, Hartoyo E, Jaya UA, Ma'roef CN, Khin Saw M, Gillis A, et al. Detection and genetic characterization of human enterovirus A71 from hand, foot and mouth disease cases in Banjarmasin, Indonesia. Southeast Asian Journal of Tropical Medicine and Public Health. 2018;49(5):820-5.
8. Zhou X, Zhu Q, Xia W, He F, Hu M, Ni X, et al. Molecular epidemiology of an outbreak of hand, foot, and mouth disease associated with subgenotype C4a of enterovirus A71 in Nanchang,

China in 2014. J Med Virol. 2015;87(12):2154-8. Epub 2015/06/11. doi: 10.1002/jmv.24288. PubMed PMID: 26058813.
9. Zhang YC, Jiang SW, Gu WZ, Hu AR, Lu CT, Liang XY, et al. Clinicopathologic features and molecular analysis of enterovirus 71 infection: report of an autopsy case from the epidemic of hand, foot and mouth disease in China. Pathol Int. 2012;62(8):565-70. Epub 2012/07/26. doi: 10.1111/j.1440-1827.2012.02837.x. PubMed PMID: 22827767.
10. Chang SC, Li WC, Chen GW, Tsao KC, Huang CG, Huang YC, et al. Genetic characterization of enterovirus 71 isolated from patients with severe disease by comparative analysis of complete genomes. J Med Virol. 2012;84(6):931-9. Epub 2012/04/14. doi: 10.1002/jmv.23287. PubMed PMID: 22499017.
11. Cordey S, Petty TJ, Schibler M, Martinez Y, Gerlach D, van Belle S, et al. Identification of site-specific adaptations conferring increased neural cell tropism during human enterovirus 71 infection. PLoS Pathog. 2012;8(7):e1002826. Epub 2012/08/23. doi: 10.1371/journal.ppat.1002826. PubMed PMID: 22910880; PubMed Central PMCID: PMCPMC3406088.
12. Wu WH, Kuo TC, Lin YT, Huang SW, Liu HF, Wang J, et al. Molecular epidemiology of enterovirus 71 infection in the central region of Taiwan from 2002 to 2012. PLoS One. 2013;8(12):e83711. Epub 2014/01/07. doi: 10.1371/journal.pone.0083711. PubMed PMID: 24391812; PubMed Central PMCID: PMCPMC3877097.
13. Jiang B, Zhang J, You X, Dong C, Cheng X, Dai X, et al. Diagnosis of hand, foot, and mouth disease caused by EV71 and other enteroviruses by a one-step, single tube, duplex RT-PCR. J Med Virol. 2012;84(11):1803-8. Epub 2012/09/22. doi: 10.1002/jmv.23391. PubMed PMID: 22997084.
14. Xu J, Wang F, Zhao D, Liu J, Su H, Wang B. Sequence analysis-based characterization and identification of neurovirulence-associated variants of 36 EV71 strains from China. J Med Virol. 2018;90(8):1310-7. Epub 2018/04/01. doi: 10.1002/jmv.25081. PubMed PMID: 29603282.
15. Duong V, Mey C, Eloit M, Zhu H, Danet L, Huang Z, et al. Molecular epidemiology of human enterovirus 71 at the origin of an epidemic of fatal hand, foot and mouth disease cases in Cambodia. Emerg Microbes Infect. 2016;5(9):e104. Epub 2016/09/22. doi: 10.1038/emi.2016.101. PubMed PMID: 27651091; PubMed Central PMCID: PMCPMC5113052.
16. Wu Y, Yeo A, Phoon MC, Tan EL, Poh CL, Quak SH, et al. The largest outbreak of hand; foot and mouth disease in Singapore in 2008: the role of enterovirus 71 and coxsackievirus A strains. Int J Infect Dis. 2010;14(12):e1076-81. Epub 2010/10/19. doi: 10.1016/j.ijid.2010.07.006. PubMed PMID: 20952237.
17. Tan CY, Gonfrier G, Ninove L, Zandotti C, Dubot-Peres A, de Lamballerie X, et al. Screening and detection of human enterovirus 71 infection by a real-time RT-PCR assay in Marseille, France, 2009-2011. Clin Microbiol Infect. 2012;18(4):E77-80. Epub 2012/02/16. doi: 10.1111/j.1469-0691.2012.03769.x. PubMed PMID: 22332991.
18. Yu H, Chen W, Chang H, Tang R, Zhao J, Gan L, et al. Genetic analysis of the VP1 region of enterovirus 71 reveals the emergence of genotype A in central China in 2008. Virus Genes. 2010;41(1):1-4. Epub 2010/03/23. doi: 10.1007/s11262-010-0472-9. PubMed PMID: 20306124.
19. Guerra JA, Waters A, Kelly A, Morley U, O'Reilly P, O'Kelly E, et al. Seroepidemiological and phylogenetic characterization of neurotropic enteroviruses in Ireland, 2005-2014. J Med Virol. 2017;89(9):1550-8. Epub 2017/01/11. doi: 10.1002/jmv.24765. PubMed PMID: 28071799.
20. Mizuta K, Abiko C, Murata T, Matsuzaki Y, Itagaki T, Sanjoh K, et al. Frequent importation of enterovirus 71 from surrounding countries into the local community of Yamagata, Japan, between 1998 and 2003. J Clin Microbiol. 2005;43(12):6171-5. Epub 2005/12/08. doi: 10.1128/jcm.43.12.6171-6175.2005. PubMed PMID: 16333123; PubMed Central PMCID: PMCPMC1317214.
21. Miyamura K, Nishimura Y, Abo M, Wakita T, Shimizu H. Adaptive mutations in the genomes of enterovirus 71 strains following infection of mouse cells expressing human P-selectin glycoprotein ligand-1. J Gen Virol. 2011;92(Pt 2):287-91. Epub 2010/10/15. doi: 10.1099/vir.0.022418-0. PubMed PMID: 20943886; PubMed Central PMCID: PMCPMC3081077.

22. Herrero LJ, Lee CS, Hurrelbrink RJ, Chua BH, Chua KB, McMinn PC. Molecular epidemiology of enterovirus 71 in peninsular Malaysia, 1997-2000. Arch Virol. 2003;148(7):1369-85. Epub 2003/06/27. doi: 10.1007/s00705-003-0100-2. PubMed PMID: 12827466.
23. Li R, Zou Q, Chen L, Zhang H, Wang Y. Molecular analysis of virulent determinants of enterovirus 71. PLoS One. 2011;6(10):e26237. Epub 2011/11/01. doi: 10.1371/journal.pone.0026237. PubMed PMID: 22039449; PubMed Central PMCID: PMCPMC3198388.
24. Huang SW, Wang YF, Yu CK, Su IJ, Wang JR. Mutations in VP2 and VP1 capsid proteins increase infectivity and mouse lethality of enterovirus 71 by virus binding and RNA accumulation enhancement. Virology. 2012;422(1):132-43. Epub 2011/11/15. doi: 10.1016/j.virol.2011.10.015. PubMed PMID: 22078110.
25. AbuBakar S, Chee HY, Al-Kobaisi MF, Xiaoshan J, Chua KB, Lam SK. Identification of enterovirus 71 isolates from an outbreak of hand, foot and mouth disease (HFMD) with fatal cases of encephalomyelitis in Malaysia. Virus Res. 1999;61(1):1-9. Epub 1999/07/30. PubMed PMID: 10426204.
26. Wang LC, Tang SQ, Li YM, Zhao HL, Dong CH, Cui PF, et al. A comparison of the biological characteristics of EV71 C4 subtypes from different epidemic strains. Virol Sin. 2010;25(2):98-106. Epub 2010/10/21. doi: 10.1007/s12250-010-3102-8. PubMed PMID: 20960306.
27. Wu Z, Yang F, Zhao R, Zhao L, Guo D, Jin Q. Identification of small interfering RNAs which inhibit the replication of several Enterovirus 71 strains in China. J Virol Methods. 2009;159(2):233-8. Epub 2009/06/06. doi: 10.1016/j.jviromet.2009.04.002. PubMed PMID: 19490979.
28. Mao Q, Dong C, Li X, Gao Q, Guo Z, Yao X, et al. Comparative analysis of the immunogenicity and protective effects of inactivated EV71 vaccines in mice. PLoS One. 2012;7(9):e46043. Epub 2012/10/03. doi: 10.1371/journal.pone.0046043. PubMed PMID: 23029378; PubMed Central PMCID: PMCPMC3460965.
29. Zhang Y, Wang J, Guo W, Wang H, Zhu S, Wang D, et al. Emergence and transmission pathways of rapidly evolving evolutionary branch C4a strains of human enterovirus 71 in the Central Plain of China. PLoS One. 2011;6(11):e27895. Epub 2011/11/30. doi: 10.1371/journal.pone.0027895. PubMed PMID: 22125635; PubMed Central PMCID: PMCPMC3220707.
30. Sun LM, Zheng HY, Zheng HZ, Guo X, He JF, Guan DW, et al. An enterovirus 71 epidemic in Guangdong Province of China, 2008: epidemiological, clinical, and virogenic manifestations. Jpn J Infect Dis. 2011;64(1):13-8. Epub 2011/01/27. PubMed PMID: 21266750.
31. Chang GH, Lin L, Luo YJ, Cai LJ, Wu XY, Xu HM, et al. Sequence analysis of six enterovirus 71 strains with different virulences in humans. Virus Res. 2010;151(1):66-73. Epub 2010/04/20. doi: 10.1016/j.virusres.2010.04.001. PubMed PMID: 20398708.
32. McMinn P, Lindsay K, Perera D, Chan HM, Chan KP, Cardosa MJ. Phylogenetic analysis of enterovirus 71 strains isolated during linked epidemics in Malaysia, Singapore, and Western Australia. J Virol. 2001;75(16):7732-8. Epub 2001/07/20. doi: 10.1128/jvi.75.16.7732-7738.2001. PubMed PMID: 11462047; PubMed Central PMCID: PMCPMC115010.
33. Wen HL, Si LY, Yuan XJ, Hao SB, Gao F, Chu FL, et al. Complete genome sequencing and analysis of six enterovirus 71 strains with different clinical phenotypes. Virol J. 2013;10:115. Epub 2013/04/13. doi: 10.1186/1743-422x-10-115. PubMed PMID: 23577963; PubMed Central PMCID: PMCPMC3669102.
34. Yip CC, Lau SK, Lo JY, Chan KH, Woo PC, Yuen KY. Genetic characterization of EV71 isolates from 2004 to 2010 reveals predominance and persistent circulation of the newly proposed genotype D and recent emergence of a distinct lineage of subgenotype C2 in Hong Kong. Virol J. 2013;10:222. Epub 2013/07/05. doi: 10.1186/1743-422x-10-222. PubMed PMID: 23822185; PubMed Central PMCID: PMCPMC3716818.
35. Brown BA, Oberste MS, Alexander JP, Jr., Kennett ML, Pallansch MA. Molecular epidemiology and evolution of enterovirus 71 strains isolated from 1970 to 1998. J Virol.

1999;73(12):9969-75. Epub 1999/11/13. PubMed PMID: 10559310; PubMed Central PMCID: PMCPMC113047.
