# Supplementary material for: Electrostatic interactions at the five-fold axis alter heparin-binding phenotype and drive enterovirus A71 virulence in mice
Source: PLoS Pathog. 2019 Nov 15;15(11):e1007863. doi: 10.1371/journal.ppat.1007863 (PMC6881073; doi:10.1371/journal.ppat.1007863)
Supplement: S1 Text — (DOCX) [file ppat.1007863.s011.docx]

**S1 Text.** **Establishment of *in vitro* blood-brain barrier model**

Primary porcine brain endothelial cells (PBECs) were cultured as reported previously with slight modifications [116, 117]. To set up *in vitro* blood-brain barrier (BBB) model, the PBECs were seeded onto Corning Transwell inserts at a density of 1 × 10^5^ cells/cm^2^. Upon cell confluency, the culture medium was changed to a serum-free medium containing hydrocortisone (550 mM). The cells were treated with CPT-cAMP (250 μM) and RO-20-1724 (17.5 μM) for approximately 24 hours to induce BBB differentiation, followed by measurement of transendothelial electrical resistance (TEER).

TEER of the PBEC monolayer was measured using a STX-100C chopstick electrode pair connected to an EVOM meter (World Precision Instruments Inc., Sarasota) as an indicator for BBB tight junction function. The first measurement was conducted approximately 24 hours post CPT-cAMP and RO-20-1724 treatment, and prior to virus exposure. TEER of a blank filter insert without cells was subtracted from measured TEER of cell monolayers, and the values multiplied by surface area of the filter insert (1.12 cm^2^) to give the final unit of Ω.cm2. All monolayers used for this study showed TEER above 100 Ω.cm^2^.

EV-A71 variants were added to the luminal (‘blood-facing’) compartment i.e. the filter inserts, and the filter inserts was incubated at 37°C. After 1-hour incubation, medium containing virus samples was removed and replaced with fresh serum-free medium with added hydrocortisone (550 nM), and the inserts were returned to the incubator. Subsequently, TEER was measured at 2 and 6 hour post-exposure. At each time point, samples were aliquoted from the luminal and abluminal (‘brain-facing’) compartments for qRT-PCR quantitation. Mock infected controls were included for comparison.
